# Swiss Cohort & Biobank – The White Paper

**DOI:** 10.3389/phrs.2022.1605660

**Published:** 2022-12-23

**Authors:** Nicole Probst-Hensch, Murielle Bochud, Arnaud Chiolero, Luca Crivelli, Julia Dratva, Antoine Flahault, Daniel Frey, Nino Kuenzli, Milo Puhan, L. Suzanne Suggs, Corina Wirth

**Affiliations:** ^1^ Department of Epidemiology and Public Health, Swiss Tropical and Public Health Institute (Swiss TPH), Allschwil, Switzerland; ^2^ University of Basel, Basel, Switzerland; ^3^ Swiss School of Public Health (SSPH+), Zürich, Switzerland; ^4^ Swiss Society for Public Health, Bern, Switzerland; ^5^ Department of Epidemiology and Health Systems (DESS), University Center for General Medicine and Public Health (Unisanté), Lausanne, Switzerland; ^6^ Population Health Laboratory (#PopHealthLab), University of Fribourg, Fribourg, Switzerland; ^7^ Institute of Primary Health Care (BIHAM), University of Bern, Bern, Switzerland; ^8^ Department of Business Economics, Health and Social Care, University of Applied Sciences and Arts of Southern Switzerland, Manno, Switzerland; ^9^ Institute of Public Health Università della Svizzera Italiana, Lugano, Switzerland; ^10^ Institute of Public Health, Department of Health Sciences, ZHAW Zürich University of Applied Sciences, Winterthur, Switzerland; ^11^ Institute of Global Health, Faculty of Medicine, University of Geneva, Geneva, Switzerland; ^12^ Epidemiology, Biostatistics and Prevention Institute (EBPI), University of Zurich, Zurich, Switzerland

**Keywords:** Swiss cohort, biobank, white paper, public health, health data, personalized health, health care, health systems

## Preamble

A large Swiss Cohort with 100,000+ representative participants of all ages is the backbone of public health related research and an essential point of reference for health systems steering, personalized health, and clinical research. The national multidisciplinary public health sciences community of SSPH+ and the national society of public health professionals—the Swiss Society for Public Health—assembled an *ad hoc* Steering & Writing Committee led by Prof. Nicole Probst-Hensch, Swiss TPH to publish a White Paper in fall 2022. This is a further complementary step in the national move toward better health data in Switzerland and specifically toward a large population-based cohort and biobank, with its many milestones, including the cohort pilot study of the Federal Office of Public Health (FOPH), largely based on the protocols of the only national population-based cohort and biobank of Switzerland (SAPALDIA) running successfully since >30 years as well as draft questionnaires of HBM4EU, Food frequency questionnaires of MenuCH, worker exposure questionnaires, and others. Some protocols have been developed specifically the cohort pilot, e.g., all blood and urine sampling protocols. Moreover, the parliamentary petitions (Motion 19.4069^1^; Postulat 21.3220^2^) call for a Children Cohort as another crucial step in the promotion of more Swiss research for the health of the youngest.

## Executive Summary

**FIGURE 1:**
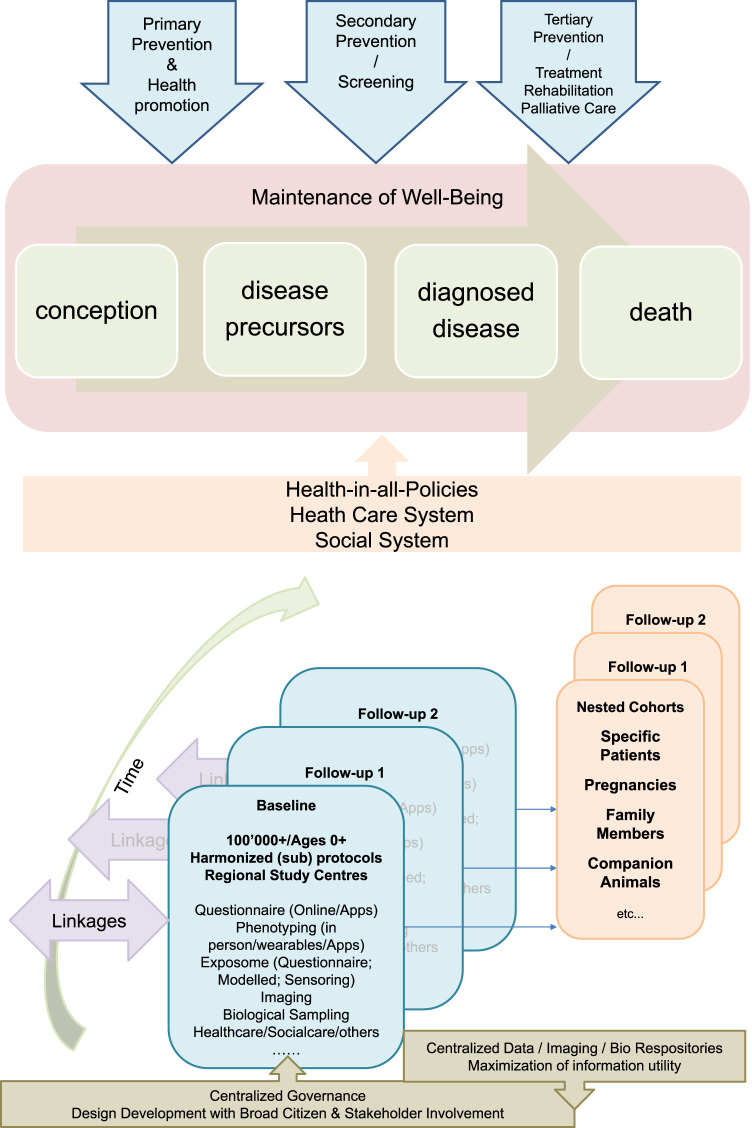
The Swiss Cohort and Biobank consisting of at least 100,000 individuals of all ages and evolving sub-cohorts will be designed in the context of a broad population and stakeholder involvement. It will be centrally governed, but implemented by regional public health research hubs, and will make use of existing and in part centralized infrastructures. It will provide relevant data, biological specimens and images to a) understand factors promoting healthy growing up and health aging towards evidence-based primary prevention and health promotion, b) identify and evaluate the utility of novel biomarkers and algorithms for risk and disease screening, and c) evaluate the utility of novel medical interventions and treatments and will thus provide the evidence-base for sustainable health and social care systems and for health-in-all policies.

A Swiss Cohort and Biobank will strengthen the development of population health sciences and of public health surveillance in Switzerland. Essential for the international competitiveness of health sciences in Switzerland, it will be interrelated and complementary to existing research infrastructure platforms, namely the Swiss Personalized Health Network (SPHN) and the National Coordination Platform Clinical Research (CPCR). The need for a Swiss Cohort and Biobank is explicitly listed in the CPCR White Paper (SAMW). Moreover, the first White Paper for the SPHN acknowledged that “in a second phase, there is a need for a large healthy population-based reference cohort”. This current White paper now prepares for the funding of the Swiss Cohort and Biobank as a sustainable national research infrastructure for public and personalized health that complements clinical research and as a longitudinal health surveillance instrument (also see Figure 1).

Switzerland has successfully implemented several internationally highly visible clinical and citizen cohort studies. However, today Switzerland is no longer able to contribute a sufficiently large population cohort to international cohort and biobank consortia which bring together long-term studies that each include over 100,000 participants. In the era where health data is the “new gold,” this will jeopardize the scientific, digital and related economic success of the country. It also means that the country lacks the longitudinal evidence-base for effective health promotion and primary prevention at all ages. Population cohorts are at the heart of global efforts that estimate population-specific disease and risk factors burden to support evidence-based policy in achieving health and wellbeing and related sustainable development goals. Finally, not having a population cohort limits the longitudinal surveillance and evidence-base much needed to evaluate and promote an evidence-based, high-value, cost-effective healthcare system and to support pandemic preparedness.

Population cohorts with associated biobanks adopting internationally harmonized core protocols are essential research and surveillance instruments for:

a) Exposome science (the primary prevention arm of precision health) towards approaching causal understanding of how social, environmental, behavioral, cultural, economic, as well as molecular factors and chemicals alone and in combination promote or hinder healthy growing up and aging. Long-term health effects of environmental factors and chemicals cannot be studied in the context of randomized trials—cohorts with integrated biobanks are the gold standard approach instead.
b) Assessing the long-term utility and cost-effectiveness of new public and personalized health interventions (e.g., physiological, imaging or molecular biomarkers for risk prediction and screening; diagnostics, treatments, and guidelines; health promotion and public health policies).
c) Evaluating the long-term impact and cost-effectiveness of healthcare system level interventions and other policy domains on health and wellbeing of people along pathways from conception to death, from healthy to disease diagnosis, regression, and progression.

Population cohorts therefore are an essential pillar of the evidence base needed for addressing major public health challenges of the 21st century, such as; population growth and future decline, demographic aging, urbanization, global warming, global trade or digitalization of society and for understanding and preventing health inequalities.

The public health challenges call for more emphasis on improving quality of life and less on extending life at any price, hence, more emphasis on primary prevention and health promotion through health-in-all-policies. In fact, to further increase healthy life expectancy, which is leveling off in many countries including Switzerland, drastic changes in the promotion of health may be needed in childhood and early adulthood, before the onset of diseases can occur. Questions of high public health relevance are: What are environmental, social, behavioral, cultural, economic, and molecular factors that maintain health and wellbeing at all ages? How can health-in-all policies promote resilience to diseases in a socially equitable manner? The long-term follow-up of individuals at different ages in the context of a cohort allows studying health trajectories with a life course perspective.

Today’s public health challenges cannot be met from within the healthcare system and by medical advances alone. We may have entered an era of diminishing returns on medical investments. The costs of high-tech medicine are a threat to the healthcare system. This calls for stringent and independent longitudinal surveillance of long-term cost-effectiveness and utility of expansive innovations. Can we turn the technological, medical and digital knowledge of the human body into meaningful improvements in population health at sustainably affordable prices and without widening the social inequity gap? Which innovations have long-term utility? These, and others, are therefore burning questions of high public health relevance towards maintaining a sustainable healthcare system.

Switzerland therefore needs its own, yet internationally harmonized, large-scale cohort for several reasons:

• For the Swiss population to benefit in the mid- and long-term from high-quality longitudinal research that captures their context-specific chronic exposures to beneficial and/or potentially damaging broad exposome factors and their impact on health and wellbeing, while taking into account individual-specific factors (e.g., social/family/work circumstances; use and perception of environment, socio-economic situation, cultural backgrounds, genetic make-up).
• For the Swiss healthcare system and healthcare providers to benefit from population-based long-term information to evaluate their functioning and to identify priorities for action and adaptation towards a sustainable healthcare system.
• For cantonal and federal public health authorities to benefit from timely evidence-based longitudinal information and human biomonitoring to plan and orient public health policies and interventions and to effectively investigate and respond to technological trends, medical innovations, as well as new environmental or pathogenic health threats.
• For cantonal and federal authorities in all domains to benefit from timely and longitudinal information on where and how to integrate health and wellbeing in their policies and planning (e.g., urban planning; sustainable food production; occupational health).
• For Swiss researchers to be internationally competitive by a) having access to and bringing to the international negotiating table high-quality open access longitudinal data, biological specimens and medical images on a large scale and b) benefitting from access to international genetic and exposome big data for providing novel insight into disease processes through comparative approaches.
• For Swiss academic career promotion in various research domains including digital and e-health to have access to rich longitudinal exposure, health and biomarker data
• For Swiss science for people (citizen science) to benefit from research conducted in close collaboration with the population to assure that 1) research priorities and conduct reflect their needs and expectations and 2) that the rights and participation motivations of data, biospecimen and imaging donors are respected
• For Swiss public-private partnerships to benefit from ample synergistic opportunities offered by a large population data, images, and biospecimens in life and data sciences and technologies

The planned cohort must recruit at least 100,000 persons of all ages from different regions in Switzerland who are subsequently followed up for at least 10 years. This makes it internationally competitive and offers longitudinal research and surveillance data for culturally and geographically different parts of the country. The design and ethical-legal background of the cohort must be flexible enough to integrate evolving novel research questions in a timely, efficient and collaborative manner. The age-range of the participants must cover the whole life-course from conception to old age. This can be achieved by age-stratified, population registry-based sampling supported by additional sampling sources for assuring adequate representation of all population groups (e.g., migrants; elderly; other vulnerable groups). The nationally and internationally harmonized core study protocol implemented in all study centers has to adhere to stringent quality guidelines, to cover broad exposure, health, wellbeing, and biomarker domains to maximize the return-on-investments made into the cohort. The study design needs to allow for age and subgroup specific study sub-protocols to meet the specific needs of funders, stakeholders, and participating research institutions. As a novel approach, the nesting of birth, family, patient, companion animal owner, occupational, and other sub-cohorts into a large Swiss population cohort should be evaluated. For example, the population-based patient cohorts which are not limited to those treated in university hospitals, can serve the clinical research platform coordinated by SAMW. For example, the integration of companion animals can support One Health approaches, e.g., the investigation of causes of cancer. For example, the recruitment of pregnancies at an early stage can support life course epidemiology with rich pre-conception and very early life data. Population cohorts are in direct and close contact with various population subgroups. In partnership with participants and with experts in social sciences, ethics and law, communication and social marketing the needs of the population and of study participants can be investigated.

Main guiding principles for the large-scale Swiss population cohort include:

• Pursuit of the best collaborative, competitive, and high quality science in the most ethical, sustainable and efficient manner possible.
• Recognition of contributions, needs, and rights of individual scientific researchers.
• Recognition of and respect for contributions, rights, needs, and privacy of study participants in close science-population collaboration.
• Sustainable funding as a research infrastructure to assure the flexibility and timeliness of data collection and design needed for research and policy—following the principles of cohorts as research infrastructures in other countries—allowing for the possibility of integrating unrestricted private funds, e. g., funding of—omics analyses.
• Local and regional public health research institutes represent the regional hubs that collect people data.
• Close collaboration and partnership between public health, different research and policy stakeholders in developing the study protocols and deciding on priorities and focus.
• Harmonization of study protocols (consisting of age-independent and age-specific basic protocols as well as of sub-protocols) with international cohorts, with relevant Swiss surveys and cohorts, and across the Swiss study centers.
• Application of information and consent procedures supporting linkage to relevant disease and administrative registries including electronic patient records in the future, and supporting national as well as global research collaboration and public-private partnership.
• Application and integration of current and emerging technologies, infrastructures, and protocols for data and biomarker collection, processing and storage in a secure and privacy-protecting manner in close collaboration with existing infrastructure and organizations, including SPHN, SAMW Clinical Research Initiative and Swiss Biobanking Platform.
• Adoption of FAIR principles for the study instruments and collected data and metadata.
• Transparent agile governance structure.

Public health is well positioned to coordinate the broad governance and stakeholder involvement needed to maximize the scientific and policy utility of a large-scale Swiss population cohort. The public health community and associated research institutes have the competence, experience, infrastructure, and the will to jointly develop and implement a Swiss large scale population cohort in close translational partnership with other scientists and with policymakers. Public health is by design a transdisciplinary domain. Implementing and coordinating cohorts is a fundamental public health task. Population cohorts are a central infrastructure for epidemiological research into disease etiology, health promotion, and health systems’ performance. Through SAPALDIA, Corona Immunitas, and the pilot phase of the Swiss Health Study, as well as more local biobanks linked to CoLaus and Bus Santé, the Swiss public health research community of the SSPH+ network has proved its ability to set-up and lead population cohort programs in a national inter-university and transdisciplinary collaboration and to assure the policy impact of research results for the benefit of people.

## Definitions

Public health is what we do together as a society to ensure the conditions in which everyone can be healthy.^3^

A population cohort is a population-based long-term study that repeatedly assesses health- and wellbeing-related aspects of individuals living in a demographically well-defined and characterized geographic area that are selected on the basis of a random process not based on disease or risk. A population cohort can focus on specific risk and health aspects or be very broad. It typically measures the participants’ physical, medical, behavioral, environmental, and sociodemographic characteristics and collects biomarkers (e.g., health measurements; biological specimens; images) once or repeatedly based on a standardized protocol including stringent quality control procedures, and follows participants forward for years. Information and biomarkers are collected repeatedly before the occurrence of clinical disease. This allows for studying the development of diseases and their causes by generating health trajectories specific to the social, economic, political and physical environment of the respective geographic area. As far as possible, recruitment of participants into the cohort aims to achieve representativeness of the source population from which participants are recruited. If the goal of the cohort research is to understand health and disease in the general population, random sampling from population registries is the designated “gold” standard, although complementary approaches are needed to ensure high external validity and sufficient sample sizes for otherwise underrepresented groups. Prospective population-based cohort studies are a crucial epidemiologic tool, particularly for identifying risk factors for diseases and comorbidities and measuring their impact, but also for the longitudinal surveillance of different health- and wellbeing-related aspects in a population as well as to evaluate the long-term impact of public health interventions. Acknowledging the complexity of non-communicable diseases and the related sample size needs, very large scale population cohorts were more recently implemented in several countries. They apply internationally harmonized core protocols and have several advantages over traditional smaller population-based cohorts. Most of these studies include 100,000 participants or more [1]. They allow for analyzing the association of combinations of risk factors with single and combined phenotypes as well as with disease subtypes with sufficient statistical power, taking complex interactions into consideration. Particularly in the context of cohort consortia that combine data from individual studies, some more rare diseases can also be studied. In case of prospectively sampled biomaterial collections, large scale cohorts enable genomics and other–omics analyses, relevant for a deeper understanding of disease development. Such cohorts are especially valuable for studying key demographic subgroups and groups of increased vulnerability with sufficient power. They can also help to refine risk modeling, identify opportunities for improved public health efforts and assess their long-term impact, examine variability in response and access to therapeutic and/or public health interventions, and identify new targets for intervention. Moreover, the infrastructure of such large-scale cohorts can be used in due time in response to public health emergencies such as the pandemic to complement existing information with complementary data needed in response to emerging challenges.

## Introduction

### Public Health Challenges of the 21st Century

The COVID-19 pandemic has brought to light the globalization of public health challenges. Viral infections, rapid spread, and the consequences of the containment measures do not respect frontiers. It also reminded us of the social dimensions of health as expressed by evolutionary biologist Richard Lewont: “Viruses and bacteria are not the causes of pandemics. They are the agent of social causes, of social formations that determine the nature of our productive and consumption lives, and in the end, it is only through changes in those social forces that we can get to the root problem of health” [2]. The same is true for many other equally or even more pressing societal, ecologic, and health issues. Major public health challenges not only share the aspect of globalization, but they also share the impact on social determinants of health and contribute to socio-economic inequity. Humanitarian crises and violent conflicts are in part the consequences of institutionally poorly addressed public health challenges, and they aggravate the consequences of the public health challenges.

**Demographic aging and population growth**—partly resulting from enormous medical and public health progress over the past decades in many parts of the world—is on the one hand leading to denser living conditions, human encroachment into pristine environments, loss of biodiversity and associated increases in the risk of zoonosis. Even though projections are for a future decrease in population growth, the pressure of the current growth over the next decades on the above aspects is of great concern. On the other hand, the disability burden due to non-communicable and age-related ailments is increasing. The aging of the population is changing welfare and institutional needs and the needs related to living conditions and social support. It puts a pressure on health and social care needs. Despite the impressive increase in life expectancy in the 20th century, life expectancy is highly variable across and within countries with a considerable socio-economic inequity gap even in high income countries [3]. According to the institutional theory of health inequalities, the welfare state as an institutional arrangement plays an important role in determining, moderating and mediating social inequalities and their health effects [4]. In part, as a result of the social inequity gap in lifestyle, environmental exposures and access to health services, all affected by policy—e.g., life expectancy has fallen in the United States and plateaued in United Kingdom, with the health inequality gap widening [5, 6]. In most cases, a person’s zip code is a stronger determinant of life expectancy than their inherited genetic code. Life expectancy at birth in Switzerland is currently one of the highest in the world. This is a consequence of a sharp increase during the 20th century. However, a gradual slowing down of this trend can also be seen.^4^

**Urbanization** leads to drastically changing physical, social, and economic environments and strongly impacts on human behavior, health, and wellbeing. In 2007, for the first time in human history, more than 50% of the global population lived in urban environments with increasing trend. This percentage is even higher (73%) in Switzerland, a country characterized by limited space and close proximity between urban and rural settings. The rapid increase in urbanization and the associated crowded living conditions increase opportunities for infection transmissions. Clearing of rural spaces and forests for the expansion of urban space increase the likelihood of contact between humans and wildlife and thereby of zoonosis.^5^ The pressure on rural and agricultural space also threatens sustainable food production. The use of plant protection products and the pressure on the agricultural workforce may increase, leading to pesticide related adverse health effects in consumers and farmers and aggravating rural exodus. Urban lifestyle, stimulated by changing living and working conditions, but also by changing cultural norms or by the influence of advertisement, increases the risk of non-communicable diseases including cancers, cardiovascular, metabolic and respiratory diseases, as well as mental and musculoskeletal disorders. Urbanity related health challenges also include, but are not limited to traffic related noise and air pollution emissions, social stress, lack of greenness, or elevated exposure to light at night. There is a strong need for changing the way we plan and manage cities in the future. Thereby contrasting effects of city densification need to be taken into consideration: while denser cities can offer more space supporting leisure time activities and social encounters, better access to facilities, less traffic-related air pollution or noise exposure, and thus better quality of life [7], they can in contrast pose a threat to the spread of infections [8, 9] or create social stress. In the small country of Switzerland, urban planning needs to take periurban and rural space into consideration, given their close proximity.

**Global warming** is an environmental threat of unprecedented dimension in need of both global and local socially acceptable solutions. Heat waves have a direct acute adverse effect on human health and wellbeing [10–13]. The direct long-term health and wellbeing effects still remain rather poorly understood. In addition, global warming has numerous indirect adverse effects on health and wellbeing. They include the rise of vector-borne diseases in so far not affected regions, the increase in extreme weather events and associated flooding, landslides, droughts, or wildfires, the aggravation of humanitarian crises, political conflicts and wars over fights for access to increasingly scarce water sources or fertile land, among others [14, 15].^6^ Global warming also has a strong impact on the economy and therefore is a major driver of **social inequity** and of **migration** [16].

**Globalization** of trade has both negative and positive effects on various health aspects [17]. Adverse effects need local monitoring and responses. Globalization of trade directly increases the risk of the global spread of zoonoses and infections. Pandemics and conflicts have brought to light the sensitivity of health relevant domains such as food supply, energy supply, as well as medication and vaccine supply to interruptions of global supply chains [18]. The spread of antimicrobial transmission is promoted by increased global trade and associated human and animal migration and travel. For example, colistin-resistant bacterial strains recently observed in high income countries are likely rooted in the wide use of colistin in Chinese livestock and transmission from livestock to humans through food [19].

**Digital transformation of societies and health systems** is a critical public health and wellbeing issue. Online life makes up an increasingly larger part of our everyday life. Digitalization changes social interactions, patient-physician relationships, medical diagnosis and treatment, business and jobs, as well as privacy concerns. It also changes the ways of epidemiology and public health. While the new technologies offer tremendous opportunities in problem solving, they can also increase dependencies (e.g., between healthcare system, physicians and private industries) and widen socio-economic and gender inequities [20–22]. In the absence of energy such as in scenarios resulting from the current war in the Ukraine, many health-related activities will be disrupted, e.g., electronic patient records and electronic patient information systems will not be disposable.

**Current demographic, ecological, social, and economic forces** will create **new priorities for public health and for future biomedical and digital innovations**. There is a pressing need to put more emphasis on improving quality of life and less on extending life, and hence, more emphasis needs to be placed on **primary prevention** and **health promotion through health-in-all-policies**. There is a growing need for a shift to value-conscious innovation instead of the “progress at any price” attitude that has dominated biomedical innovation until now [23]. The innovation driven rapid increase in healthcare expenditures is not sustainable for the healthcare systems or for individuals and families. Therefore, the long-term utility and cost-effectiveness of innovations must be assessed and discussed in relation to diverse perspectives (e.g., society, insurance, governments) to support evidence-based policy decisions towards sustainable health-in-all policies.

### Diminishing Returns in High-Tech Medicine—Shifting Focus on Public Health

There have been a few key innovations increasing quality of care and decreasing the cost of care with positive effects such as reduction in mortality and morbidity rates, relief from pain, or improvement of care that patients and people desire. Examples are antibiotics, diuretics or some vaccines. Most innovations, however, increase both quality of care and costs. Their effect on value (changes in quality relative to changes in costs) depends on the relative sizes of these increases. In a value-conscious environment, some of the most popular current and future high-tech innovations would meet a reasonable value standard, but many probably would not [23].

Early evidence suggests we may have entered an era of diminishing returns on medical investments. The gains attributable to personalized cancer medicine, where personalized treatments are most prevalent, may not achieve the promises made [24], although more stratified evidence is needed [25]. Early data for 71 cancer drugs consecutively approved between 2001 and 2012 suggested that their overall contribution to patient survival was only 2.1 months [26]. Precision oncology has had some major successes in the meantime. For example, imatinib has a 95% response rate in patients with chronic myeloid leukemia and extends quality-adjusted life by about 9 years. Yet, a study estimated that only 8% of patients with cancer are eligible for precision medications approved as of January 2018 and only 5% would actually benefit from them. Even among patients who respond, incremental survival provided by many drugs is still measured in months and adds up to hardly a full year. Partly because of cost-effectiveness concerns, of the 54 new anticancer drugs launched between 2013 and 2017, only 80% were available in Germany by the end of 2018 and only 69% were available in France (96% were available in the United States) [25].

Investments into high-tech medicine have to be carefully balanced against public health investments to assure that the social inequity in health does not widen. In the past century, public health systematized sanitation, improved food and water safety, expanded understanding of diseases, developed powerful prevention and treatment tools such as vaccines and antibiotics, and expanded capability in epidemiology and laboratory science; together making important contributions to public health. Iodine fortification of food was identified as an effective public health intervention preventing against iodine deficiency. Switzerland was among the first countries to introduce iodized salt in 1922 and the public health program established over the years has been an international role model for the partnership between government, academia, and the salt industry [27]. In the late 1970s the National Research Program 1A on Primary Prevention of Cardiovascular Disease in Switzerland assessed the efficacy of community health education in reducing cardiovascular risk factors in whole population groups. These days, Switzerland belongs to the countries with the lowest prevalence of hypertension globally due to its primary prevention and hypertension treatment efforts [28]. This progress means that comprehensive public health protection—from both, effective primary prevention and science-based medical treatment—are possible for the general population [29].

A longer life has long been one of the central goals of investments in public health and medicine. However, it is questionable whether extension of lifespan should still be a top priority. Advances in medical care contributed less to overall lifespan than did advances in hygiene, food safety, and clean water, decreases in infectious diseases, and improvements in environmental conditions and living standards [30]. Advances in medical treatment, particularly at older age, are increasingly unlikely to provide substantial gains in healthy human longevity. It is unlikely that the high-tech approaches of today will replicate the successes made in public health and the medicine of the 20th century. Human lifespan may likely have an unalterable biological limit of around 120 years. Even though the number of centenarians has increased considerably in the past decades, very few people reach an age beyond 105 years and so far, the oldest human being reached the age of 122 years [31–33]. To maintain a high life expectancy and to diminish socio-economic differences in life morbidity and expectancy, drastic changes in the promotion of health may be needed in childhood and early adulthood. Age-related diseases have their roots in early childhood and adolescents and evolve over time. Unhealthy behaviors are present from childhood to adulthood, as are geographical, socio-economic, and ethnic related differences in health inequalities [34, 35].

It is timely to shift a relevant percentage of resources away from the search for life-extending therapies. A shift in focus and investments is needed—toward identifying strategies that improve quality of life overall and compress illness at the end of life—a shift away from medical research and even end-of-life medical care toward the same social, cultural, and political factors that successfully prolonged life in the last century [36, 37]. Doing so would also mean that conditions that affect decades of a person’s life (such as arthritis, autism, macular degeneration, dementia, and poor mental health) and their prevention would have priority over end-of-life illnesses, such as end-stage cardiac disease and many types of cancer [37].

Preserving quality of life throughout the lifespan is a fundamental public health goal. However, since the second half of the last century, public health has been significantly and increasingly underfunded almost everywhere relative to healthcare spending. For example, the United States has made paltry investments in upstream, non-medical determinants of health, such as social services, education, transportation, environmental protection, and housing programs. This lack of investment has had detrimental effects on population health [29]. The healthcare system in Switzerland is of high quality, but also of high costs. Spending on prevention and health promotion remains more marginalized.

The new public health vision now recognizes that public health cannot be improved from within the healthcare system alone and by medical advances only. Cross-sectorial action at the global, national and community level is needed to further improve population health [29].

Testing of the utility of and access to high-tech and precision medicine is also a fundamental public health task and requires population-based data. Will patients finally live longer and healthier lives in the era of personalized medicine? Will society be able to afford it? Do personalized diagnostics reach those in need? The precision medicine era is a test of the health system and the biomedical and digital innovation system. Can we turn the revolution in knowledge of the human body into meaningful improvements in population health at appropriate prices? The outcome of this test is immensely important for society [25].

### The Relevance of Actionable Public Health Data

To meet the public health and health systems challenges with the relevant evidence-base, the availability of timely, reliable, granular-level, objective, and actionable data is needed and must be made accessible to policymakers and communities. Data must allow for surveillance and for research into broad health and wellbeing determinants in their full complexity. Data must also encompass clear metrics to document success in public health practice to guide, focus, and assess the impact of prevention, screening, treatment, and rehabilitation initiatives. Metrics must encompass those that are assessing and targeting the social determinants of health and enhancing equity. The public and private sectors should work together to enable more real-time and geographically granular openly accessible data to be shared, linked, and synthesized to inform action, but public concerns and technical challenges related to protecting data security and individual privacy need to be addressed first [29].

The relevance of data for addressing the urgent public health challenges and for measuring the status and progress in population health is generally well recognized. As in many other countries, Switzerland has established numerous important health-related surveillance instruments, for example mortality and disease registries, accessible medical records in the context of the Swiss Personalized Health Network, communicable disease reporting, a regularly repeated national health survey, or continuous air pollution and pollen monitoring. The value of longitudinal health surveillance and of studying disease trajectories is well recognized for persons living with certain health conditions. The Swiss HIV Cohort Study, representative of the HIV epidemic in Switzerland, is following up its registered participants and has contributed important and meaningful evidence for the optimization of patient care, to the reduction of HIV transmission, to insights into novel HIV treatments, pathogenesis, co-infections, immunology, and virus—host interactions.^7^ The Childhood Cancer Registry of Switzerland records cancers in children and adolescents and investigates retrospectively and prospectively factors influencing disease incidence, disease treatment and progression, as well as the long-term wellbeing of childhood cancer survivors.^8^

The COVID-19 pandemic, with its imminent need for actionable public health data has brought to light limitations in efficient access to and linkage of relevant health data, both in Switzerland and in most countries abroad. In response to interventions by members of the Swiss parliament in 2021 for an improved health data situation, Switzerland is now preparing for a data strategy that allows for more efficient use and linkage of health data in order to support an efficient healthcare system of high quality. The health policy strategy of the federal council in Switzerland for the period 2020–2030 has in fact put an important emphasis on the relevance of data even more generally.^9^ Health2030 also puts a lot of emphasis on assuring a life of the Swiss population (citizens) in full health, thereby not only focusing on the healthcare systems and end-of-life investments, but also on cross-sectorial health promotion.

Also in the United States, the Centers for Disease Control and Prevention’s “Climate-Ready States and Cities Initiative” not only recognizes the important role of public health in the surveillance of, and dealing with, epidemics, but also recognizes the increasingly important role of public health agencies in protecting communities during times of emerging environmental challenges due to climate change. With evolving environmental health concerns, environmental public health tracking data priorities will require strategic updates to continue informing public health decision-making at all levels of government [38].

Addressing the gap in data is also among the most urgent public health priority in low- and middle-income countries. Take the example of India, where only 21% of all registered deaths had a medically certified cause of death available in 2019 [39].

As health issues not only need data directly from the health sector, an urgent public health need is also inter-sectoral initiatives towards data integration from different policy domains, including but not limited to data on social, economic, and ecological circumstances. To allow for cross-sectorial data linkage, there is an urgent need for a unique identification number for people living in Switzerland allowing for linkage under tightly regulated conditions.

For public health relevant data to have an impact on political decisions and on population health, a close collaboration and dialogue between academia and policy is needed. Policymakers needed to understand and respect the need for the best scientific approaches; scientists need to understand the policy needs for understanding not only whether a policy works, but also why a policy works. Both sides need to develop a joint deep understanding of how to design evidence-based and impactful new policies [40]. As an example, various stakeholder perspectives, expectations and needs must be met with relevant data including health data for the governance of plant protection products. Towards that goal and for a sustainable transformation of Swiss agriculture, the Swiss National Science Foundation funded the TRAPEGO project that produces evidence in an inter- and transdisciplinary manner involving health and political scientists, agronomists, environmental scientists, decision and media analysts, and transdisciplinary scientists. “In this research, we follow the basic assumption that systematic, targeted, and timely evidence and information about pesticide exposure and risks, about alternative farm practices and policies has an impact on peoples’ attitudes towards pesticide use and regulation”.^10^ The Horizon 2020 funded HBM4EU science-to-policy project^11^ “is coordinating and advancing human biomonitoring in Europe and so provides better evidence of the actual exposure of individuals to chemicals. In addition, we provide a robust interpretation of human biomonitoring data and the possible impact of chemical exposure on human health, using the most up to date scientific tools. HBM4EU partners effectively communicate results to policymakers, ensuring their exploitation in the design of new chemicals policies and the evaluation of existing measures”.

**Switzerland**–despite being a high-income country—shares many of the data gap challenges of other countries. The data poverty arises in part from a lack of efficiently linkable national and representative data on relevant health outcomes and determinants, most strikingly in children and adolescents (Nationaler Gesundheitsbericht), and from the fragmented health systems in our country. The independent evaluation of the crisis management of the COVID-19 pandemic recently identified better availability of health data as an essential priority for future pandemic preparedness.^12^

Public health research is used to collaborate across cantons and disciplines and to collaborate with policy and meet policy needs with data. It was the public health community, which demonstrated its ability and willingness to join forces for providing population-based evidence on the course of the infection and vaccination and on broad societal outcomes of the pandemic and its containment measures in the context of the **SSPH+ Corona Immunitas** program [41].

Beyond the time of the pandemic, the data poverty of the country also jeopardizes the international competitiveness of Swiss public and personalized health research. It limits assessment of policy priorities and impact and is therefore also lacking for the steering of the Swiss healthcare system toward a sustainable, cost-effective, and equitable system. Data is also lacking as evidence for providing the much-needed health-in-all policy priorities.

### Population Cohorts for Research on Healthy Growing Up and Healthy Aging

Long-term studies offer the opportunity to characterize individual participants’ genetic, behavioral, psychological, societal, cultural, political, and environmental context and to assess the independent predictive effects of single factors or mixtures/clusters on sustainable wellbeing, health, and on the incidence of specific diseases or co-morbidities. To address the full complexity of healthy growing up and aging these long-term studies need to be broad and large. Only sufficiently sized, broad long-term studies allow examining exposures in the broad sense and their health and wellbeing consequences in a time-resolved manner. To sort out the causal role of factors and their interactions, it is necessary to characterize the temporal sequence of exposures and consecutive responses under real life conditions [42]. The long-term perspective on health trajectories in relation to disease risks is needed in the light of the fact, that many non-communicable diseases (NCDs) risks in particular have decade long latencies between exposure and disease symptom occurrence.

Many diseases arise from behavior and environment induced molecular insults that accumulate over the course of life. Life course epidemiology asks for cohort data from different age groups with repeated assessments over years to allow investigating exposure with long latencies and susceptible time windows and to allow studying behavioral and health trajectories. Cohorts covering different age groups ranging from early life to old age are an efficient solution for providing answers to many of the public health challenges in a timely manner and from a life course perspective. On the one hand, a birth cohort running over a follow-up period covering human life span would allow investigating lifelong risk and disease trajectories. Yet, it cannot provide timely answers on adverse health risks with a very long latency. On the other hand, an adult cohort allows for investigating disease trajectories and their determinants in age groups most affected by chronic diseases and associated healthcare expenditures. However, they cannot provide the data and evidence needed to understand determinants of childhood diseases and wellbeing, such as the impact of climate change or of a digital society on the current young generation.

Observational epidemiology, despite being the gold standard for studying disease etiology, is inevitably challenged by various sources of bias arising from loss to follow-up, exposure and disease misclassification, confounding, and non-generalizability of results. Yet, statistical methods combined with methods of high-tech medicine, digital technologies, and personalized health offer novel instruments for better approaching causal understanding in the context of observational cohorts. In particular, instruments for improved characterization of the external environment (e.g., satellite data, wearables, and sensors), biomarkers and imaging provide opportunities to better approach causal understanding of disease risks and processes. They do so by better understanding correlations between exposures, by decreasing exposure misclassification, by decreasing relevant disease and phenotype misclassification, by providing early disease endpoints, and by offering insights into molecular pathways mediating exposure-disease associations in a time resolved manner.

Recognizing that the genetic background of a person in most cases contributes much less to disease risk than external and in part modifiable exposures, the exposome concept was developed to parallel the genome concept and as a domain of precision health research. The exposome concept represents an individual human’s “life-course environmental exposures (including lifestyle factors), from the prenatal period onwards” [43]. It advocates a shift toward more comprehensive characterization of exposure, aiming to raise the prioritization of exposure risk factors to a comparable level as for genetic risk factors. In the exposome era—a domain of precision health research—epidemiologic research has moved far beyond, but importantly still includes questionnaire-derived information. The sharing of data from pictures, mobile phones, global positioning systems (GPS), wearables and sensors, or from medical sources between cohort participants and researchers allow unprecedented in-depth characterization of study individuals’ broad exposures over time and space that includes assessment of typical exposure clustering, occurrence of mixtures, and exposure correlations [42].

Recognizing the critical role of underlying endogenous processes in the continuum from exposure to disease, practical application of the exposome concept also includes in-depth biological characterization across molecular–omics layers to better understand mechanisms underlying diseases. As a result, the exposome was further elaborated to encompass the associated biological responses to exposures which are vital to understand environmental influence on human health [44]. Building upon this, Miller and Jones redefined the exposome as “the cumulative measure of environmental influences and associated biological responses throughout the lifespan, including exposures from the environment, diet, behavior, and endogenous processes” [45].

Biological samples accessible in a large number of healthy cohort participants (e.g., blood fractions, urine, stool, saliva, and exhaled breath) prospectively collected, processed, and stored in biobanks under high quality conditions before disease occurrence can offer insights into biological disease pathways in line with the meet-in-the-middle concept [46]. According to the meet-in-the-middle concept, molecular changes that are on the one hand predictively associated with an exposure and that are on the other hand themselves predicting disease occurrence, are informing on biological pathways mediating the exposure-disease association. Single molecules stemming from external chemical exposure (e.g., pesticides in urine) can be linked to future disease occurrence in the context of human biomonitoring embedded into cohorts. Thus, biobanks as an essential part of longitudinal cohorts allow studying molecular pathways and hallmarks mediating the association between health risks and disease development [47, 48]. The same is true for prospectively collected images and “image-banks” and imaging features can serve as early effect biomarkers. Biological understanding and understanding of pathways in a time-resolved manner are key aspects of causal inference. Under certain conditions and in the context of genetically determined risks only (e.g., obesity and addiction) Mendelian randomization even offers the equivalent of risk randomization in the context of an observational cohort based on genetic data and allows direct testing of the causality of associations under certain conditions [49, 50].

Biomarkers, derived from laboratory analysis of biological samples or from static or functional imaging of tissues and organs not only offer insight into biological fingerprints related to preclinical effects and to health risks, but additionally allow for a refined disease classification. They thereby serve to decrease misclassification of health and disease endpoints in epidemiological studies and therefore increase the likelihood of observing true associations.

Yet, many exposures do not elicit readily observable biochemical responses. Some exposure related responses are better characterized by cellular processes or biosignals indicative of, for example, behavioral and emotional responses [42]. Molecular characterizations need to complement, and be complemented with, repeated structural and psychosocial characterization at individual and macro-level [48], such as evidenced by the repeated deep characterization of participants of the ABCD study investigating the brain and cognitive development of adolescents.^13^ The greater integration of psychosocial factors and sociological expertise has been advocated to shape the development of exposome studies. This is critical to assure that multi-omics signals of limited size and public health relevance do not get more weight than the recognition of overarching factors underlying health disparities, which are often unrecognized or not deemed modifiable in public health strategies (e.g., economic and socio-economic marginalization) [42, 51]. However, multi-omics and imaging approaches can in fact add to the understanding of the biological correlates of health disparities [52].

Observational and population-based longitudinal and long-term studies are the methodological gold standard for public health and epidemiologic research that supports populations in growing up and aging healthy and well. Indeed, population cohorts are at the heart of global efforts that estimate population-specific disease and risk factors burden to support evidence-based policy in reaching health and wellbeing in a socially equitable manner and relevant for reaching sustainable development goals. Countries that lack large-scale population-based cohorts, such as many low- and middle-income countries, but also Switzerland, are faced with uncertainty in estimating their precise disease and risk factor burden, as prevalence and relative risk estimates have to be estimated based on evidence abroad. This is limiting for Switzerland with its diverse geographic and cultural contexts.

Chronic and complex factors influencing health and wellbeing over the life course cannot be randomized to humans, both for practical and ethical reasons. New chemicals (e.g., pesticides) entering the market can by definition only be assessed for long-term health effects in humans in the context of post-marketing surveillance. This highlights the need for observational approaches toward identifying health risks or benefits with long-term effects.

Modern technology ranging from satellite data to wearables, sensors, and apps and including–omics biomarkers, imaging technologies, and integrated psychosocial factors allow for a new era of precision epidemiological and public health research. **Exposome science—a domain of precision health research—**conducted in the context of large population cohorts with associated biobanks and linked to profiling tissues and organs with the help of imaging is offering novel opportunities for approaching causal understanding of disease risks and pathways.

In parallel to personalized medicine and genetic research, the realization of the exposome concept in research also poses many challenges. There is a certain risk that the overpromises made in genetics and personalized medicine are repeated in exposome research. Yet, the concept makes optimal use of novel research instruments, brings to light challenges previously ignored by many smaller scale and focused epidemiological studies (e.g., independent studies each assessing one risk factor effect on one health outcome while not adjusting for multiple testing across studies), stimulates collaborative research towards sufficient statistical power, and formulates precisely the goal of research into the understanding of modifiable chronic disease risks. Even though the ultimate goal of causal inference with regard to chronic risk effects may rarely be achievable by epidemiological cohort research alone, keeping this goal in mind is likely to improve methodological approaches as well as interpretation of results, and stimulates transdisciplinary exchange, which is needed for decision taking.

### Population Cohorts for Longitudinal Public Health Surveillance

Besides being the gold standard research instrument for risk and protective factors with chronic effects on disease occurrence, longitudinal cohorts are also important surveillance instruments for assessing the long-term impact of policies, treatment guidelines, or medical innovation on individual’s health and behaviour or for assessing the trajectories of health symptoms or diseases that exhibit temporal fluctuation.

During the COVID-19 pandemic, existing large scale citizen (population-based) cohorts such as the German National Cohort much like the newly established Corona Immunitas cohorts were able to provide insight into the course and determinants of the infections, the development of seroprevalence, the course and determinants of vaccination behavior, the adherence to and the impact of protective measures such as mask waring or social distancing, and the impact of containment measures on behavior, wellbeing and mental health trajectories [53–59]. If large cohorts exist, they can be used for very efficient evaluation of the course of the pandemic.

With regard to chronic diseases, cohort studies allow assessing for example patterns of reach and participation in screening and their impact on the longitudinal course of health [60]. Population-based (citizen) cohorts allow assessing trajectories of health phenotypes and symptoms such as depressive and mental health symptoms [61]. The association of such longitudinal disease and symptoms trajectories with medical care including telemedicine or the use of novel technologies can be assessed.

In the case of longitudinal surveillance, population-based cohort studies have the advantage of having the whole healthcare system under surveillance and not just individuals treated in centralized university hospitals. This is of particular relevance in Switzerland in the absence of broadly accessible ambulatory care data. Thus, population-based cohort data also provides primary care physicians with an important evidence-base on the long-term population health impact of their care activities.

## National and International Population Cohorts

Many of the global public health challenges can no longer be resolved by countries in isolation. Globally harmonized, yet country-specific data are needed for evidence-based global and locally adapted solutions. In addition, cross-sectional surveillance instruments are not sufficient in supporting sustainable policy impact on population health and wellbeing.

### Population Cohorts in Switzerland

Switzerland has only few population cohorts with a broad exposure and health focus to date (e.g., **SAPALDIA** (sample size/age at baseline in 1991: 9,651/18–60 years [62]); **CoLaus** (sample size/age at baseline in 2003: 6,188/35–75 years [63]); **BusSanté** (started in 1993 with yearly enrollment of 1,000 participants amounting to >20,000 participants/age 20–74 years [64]). The SAPALDIA cohort is the only Swiss-wide population-based (citizen) cohort with associated biobank and a broad healthy aging focus. Up to date, no child and adolescent cohort with a broad health focus has been established.

The available Swiss citizen cohorts have provided important and public health relevant research and surveillance evidence. For example, the **SAPALDIA** cohort is among the internationally renowned cohorts investigating the long-term effect of air pollution. Early baseline evidence on the cross-sectional association between air pollution levels and poor respiratory health contributed to the introduction of air quality standards for particulate matter in Switzerland. Subsequent longitudinal evidence demonstrated that the resulting improvement in air quality benefitted individuals’ respiratory health [65], particularly in non-obese participants [66, 67]. SAPALDIA was among the first studies to show a link between air pollution and diabetes and produced insights into pathways by which transportation noise impacts on health [68–70]. Due to the early integration of genome-wide information based on the visionary SAPALDIA biobank, molecular pathways mediating adverse health effects of air pollution could be studied [71]. SAPALDIA was leading the first publications on genome-wide and epigenome-wide associations with lung function [72, 73] and contributed to genome-wide meta-analyses of several additional phenotypes, including allergies and renal function. Due to rich exposure and phenotype assessment combined with biomarker data, SAPALDIA is participating in the largest exposome research initiatives funded by the EU [47, 74]. The **CoLaus** cohort has a strong focus on cardiovascular and metabolic phenotypes and their risk factors, including on the link between mental health and cardiovascular disease [75]. CoLaus has very rich clinical data due to its close collaboration with clinical domains for deep phenotyping of participants (e.g., sleep—HypnoLaus [76]; mental health—PsychoLaus [77]; respiratory health—PneumoLaus [78]). CoLaus developed and validated clinical tools for screening and diagnosis, e.g., a simple screening tool for obstructive sleep apnea syndrome in depressive disorders as well as risk scores for cardiovascular disease and diabetes [79–81]. CoLaus is also contributing to various genome-wide association studies on different behaviors and diseases, most importantly metabolic diseases and anthropometric traits, and is investigating the utility of polygenic risk scores [82, 83]. **Bus Santé**, which evolves as a cohort from annual cross-sectional surveys in Geneva, has produced important trend information on health aspects, including on the small scale geospatial evaluation, of the impact of policies such as mammography screening, tobacco smoking ban, or nutritional guidelines [64, 84–86]. Both, CoLaus and SAPALDIA contribute important data for deriving reference values for lung function [87].

The administrative Swiss National Cohort (SNC) is a long-term, population-based multipurpose cohort and research platform. The current version of the SNC is based on census data from 1990 to 2000 that were linked to mortality, life birth, and emigration records until 2015, and to the newly introduced register-based census and annual structural surveys from 2010 onward. The Swiss National Cohort lacks direct contact with its participants and has no associated biomarker or imaging data. Importantly though, it complements deep phenotyping cohorts with biobanks by providing large sample size for studying associations in particular with mortality. The SNC enables research in a wide range of Public Health subjects, in particular in combination with other longitudinal or environmental data.^14^ SNC has contributed important evidence on the association of various environmental, occupational, and health systems factors with all cause and cause-specific mortality and has brought to light the broad socio-economic health disparities in mortality in Switzerland [88–92].

An additional but more focused Swiss-wide cohort is the SSPH+ Corona Immunitas program. It was initiated in 2020 to study in a comparative manner the development of SARS-CoV-2 seroprevalence, vaccination status, and adherence to hygiene measures as well as the societal impact of the COVID-19 pandemic containment measures across Switzerland [41, 55]. The Corona Immunitas program has not been established with the goal of a long-term cohort, except in certain cases such as the COVCO-Basel [56] or Corona Ciao cohorts [93], which were nested into the Corona Immunitas program. Yet, the Corona Immunitas program has demonstrated the ability and willingness of the Swiss public health research community to collaborate and establish a Swiss-wide cohort applying a harmonized core protocol while at the same time offering opportunities for additional data and biosample collection according to the specific research interest of each participating institution. The Corona Immunitas as of 2022 has included over 50,000 study participants, assessed in the context of over 40 studies and in collaboration between 14 participating universities and health organizations. The program was financed through unrestricted public and private funds governed by the Swiss School of Public Health SSPH+^15^.

SAPALDIA remains for now the only Swiss-wide population biobank and is the only cohort that can provide a sufficiently narrow genetic profile for the Swiss population based on the visionary set-up of a high-quality biobank in 2001 linked to a research promoting consenting procedure. Through SAPALDIA, Corona Immunitas and the pilot phase of the Swiss Health Study, as well as more local biobanks linked to CoLaus and Bus Santé, the Swiss public health research community of the SSPH+ network has proved its scientific excellence and its ability to set-up and lead population cohort programs in a national inter-university and transdisciplinary collaboration.

This expertise and network must now be applied to setting up a large population-based Swiss citizen cohort and biobank. It is true that the existing Swiss cohorts have enabled research and surveillance on a wide range of public health and research topics. Yet, the available population-based cohorts with biobanks do not cover the full geographic and cultural diversity of Switzerland. Their study protocols have not been harmonized. They do not cover all age groups, with data on children lacking. Most importantly, their sample sizes are insufficient these days to allow studying the full complexity of disease etiology as is possible in the modern era of epidemiology. Modern day epidemiology makes use of broad high technology methods and big data to capture the external and internal exposome. It takes place in the context of international cohort consortia. The lack of a large population-based cohort and biobank now limits the international competitiveness of public and personalized health research in Switzerland. If Switzerland can bring its own large cohort into these consortia, win-win partnerships can be created and the excellence of Swiss researchers in the fields of personalized and public health can be maintained. Besides, data from a large Swiss cohort and biobank provides an insight into the Swiss-specific context, something that cannot be replaced by cohort data from abroad.

### International Population Cohorts

Beginning with the population-based Framingham Cohort Study, which follows-up from 1948 on a few thousand volunteers and has fundamentally shaped policies towards the prevention of cardiovascular diseases [94], much larger prospective cohorts including hundreds of thousands of subjects were launched in different countries, such as the Nurses’ Health Study [95], the One Million Women Study [96], the UK Biobank, deCode in Iceland [97], the Biobank Japan [98], the Kadoorie Study of Chronic Disease in China [99], the Norwegian CONOR Consortium [100], the EPIC European Prospective Investigation into Cancer and Nutrition [101, 102], or LifeLines in the Netherlands [102]. Data from these cohorts have importantly shaped guidelines and policies, ranging from nutritional guidelines to public health and clinically relevant guidelines on the long-term use of exogenous hormones to environmental guidelines, to name a few.

Other very large population-based cohorts with hundreds of thousands of participants have more recently been implemented in different countries, such as the German National Cohort [103], the Constances Cohort in France [104], LifeGene in Sweden [105] and the Cartagene Cohort in Québec, Canada.^16^

Most of the large cohort studies to date focus on adults, aging, and chronic diseases diagnosed later in life, although next generations linked to families have been recruited (e.g., Nurses’ Health Study). Fewer large prospective studies also focus on disorders that emerge early in life, in some instances during infancy or early adolescence. LifeLines in Holland [102] is a three-generation population-based study with a household recruitment approach. The Norwegian Mother and Child Cohort study (MoBa) [106], ALSPAC (the Avon Longitudinal Study of Parents and Children) [107], and the US National Child Study [108] have followed pregnant mothers from early pregnancy and their offspring throughout childhood, whilst other prospective birth cohort studies were brought together under the Global Asthma and Allergen European Network (Ga2len) [109]. The Southampton Women’s Survey [110] is of particular interest because it collected parental exposure data before the pregnancy and thus could assess associations to perinatal and infant outcomes. Pre-pregnancy exposure data are likely to be valuable in long-term studies of chronic disease later in life. LifeGene is designed as a prospective cohort study with an infrastructure that allows repeated contacts of study participants approximately every 5 years, and short follow-ups annually. Recruitment of index people aged 18–45 years who are invited to include their household members (other adults and any children) increases the opportunity to involve young couples prior to and during pregnancy, allowing for a study of children born into cohort with complete pre- and perinatal data from both the mother and father. Other types of event-based sampling (i.e., data collection initiated as a result of a relevant event, such as an accident or influenza) is a key feature of LifeGene [105].

Cohorts focusing *a priori* on the recruitment of children also exist. Among the noteworthy child cohorts are the KiGGs study in Germany [111], CHILD cohort in Canada,^17^ Copenhagen Child Cohort [112], Growing up in Ireland-Study,^18^ and the US ABCD study.^19^

### International Harmonization of Large-Scale Cohorts and Biobanks

Large cohort studies involving hundreds of thousands of participants have been established or launched in several regions worldwide—so called “Mega Cohorts.” Such cohorts provide great value for studying diverse populations and key demographic subgroups, rare genetic variants and exposures, as well as complex gene-environment interactions with sufficient statistical power. Each cohort is constrained, however, by its size, ancestral origins, and geographical boundaries, which limit the subgroups, exposures, outcomes, and interactions it can examine. Ensuring data interoperability across large cohorts provides a vast digital resource of diverse data to address questions that none of these cohorts can answer alone, enhancing the value of each cohort and leveraging the enormous public investments made in them to date [1].

Collaboration among cohorts from different global settings offers numerous benefits [1]. Identification and phenotyping of carriers of loss-of-function alleles in nearly every human gene (“human knock-out project”) is theoretically feasible if several million genome sequences are available for analysis and linked to detailed genotypic and broad phenotypic data. Assessment of rare genetic variation would be greatly enhanced if the research participants who donated these samples are available for, and accepting of, re-contact and in-depth study. Yet, relevant scientific questions to be addressed by collaboration across cohorts are not limited to studying rare exposures and outcomes. It can broaden the exposure and interaction range, which provides novel understanding of dose-response curves and risk pathways. Multi-national analyses of global health problems such as obesity and exposure to toxic substances such as alcohol and chemicals or pollutants could identify generalizable approaches for addressing global threats to public health. Context-specific analyses of the local relevance of risk factors could better inform global burden of disease estimates and assess what determines “health” in different settings. Country- or cohort-specific risk predictions using standardized methodology could also be compared with a goal not only of producing more generalizable risk estimates but also of recognizing when tailored predictions are more appropriate.

In addition to addressing specific research questions through international cohort collaboration, invaluable contributions to harmonized research methodology have been developed by a consortium motivated to develop procedures that are readily disseminated and implemented. The methods that are developed, available and continuously updated include: 1) phenotyping methods for a wide array of health outcomes using algorithms based on health record systems and other sources; 2) systems to facilitate and encourage funding for long-term follow-up; 3) novel methods (such as digital health technologies, data linkage, and large-scale imaging) for characterizing exposures, defining outcomes, and visualizing and managing data; 4) best practices for communicating results to participants shared and optimized by comparing outcomes of differing approaches in different cultures; 5) methods of meta-data and for data sharing to maximize use of cohort and biosamples; 6) support for cohort-wide bio-sample analysis and data deposition to minimize sample wastage from inefficient case-control analyses and limitations arising from batch effects; 7) development of population-specific genotyping arrays and imputation algorithms based on whole genome sequencing of specific reference populations; and (8) decrease of per-sample costs of genome sequencing and other—omics (e.g., transcriptomics, proteomics, metabolomics, etc.) through efficient processing of millions of samples. Close partnerships are needed with developers of novel assays to determine when assays are ready to be applied to millions of specimens; cohorts can work iteratively with developers to improve these assays.

Citizen cohort and biobanks with a sample size of 100,000 participants and more are the new gold standard as epidemiology and public health research has recognized the relevance of big sample size and international collaboration for elucidating the factors contributing to healthy growing up and healthy aging in their full complexity.

Leaders of large-scale cohorts, most of them from high income countries, have come together to form the International Hundred Thousand Plus Cohort Consortium (IHCC) [1]. IHCC comprises more than 60 cohorts from more than 30 countries from across the world involving roughly 30 million participants. Collaborative efforts to date have focused on developing a queryable cohort registry and data sharing platform, identifying and piloting high-priority scientific projects, and fostering collaborations.

The IHCC has as its aim “to create a global platform for translational research—cohort to bedside and cohort to bench—informing the biological and genetic basis for disease and improving clinical care and population health”.^20^ The member cohorts aim to recruit 100,000 participants or more, are disease-agnostic, have available biospecimens, and have longitudinal follow-up activities. The consortium is driving personalized and public health research globally. Countries around Switzerland contribute with cohorts such as NaKo (Germany), Constance (France), UK Biobank (United Kingdom), Danish National Birth Cohort (Denmark), East London Genes and Health (United Kingdom), EPIC (numerous countries, but not Switzerland), EpiHealth (Sweden), Estonian Genome Project (Estonia), Generations Study (United Kingdom), Genomics England (United Kingdom), Million Women Study (United Kingdom), Northern Sweden Health and Disease Study (Sweden), Norwegian Mother and Child Cohort Study (Norway), and Vorarlberg Health Monitoring and Promotion Program (Austria). It is important that this consortium will also stimulate and support mega-cohorts from low- and middle-income countries in the future.

Switzerland has international renowned expertise in establishing, maintaining and scientifically exploiting population-based (citizen) cohorts and biobanks. Yet, it is no longer able to contribute a sufficiently large cohort to the international cohort and biobank research community consortium as of today. In the era where health data is the “new gold,” this will jeopardize the scientific and also digital and economic success of the country. Current investments into the structured description of data and biosamples of existing Swiss cohorts are an important preparatory step for making data of a future large population cohort interoperable with cohorts abroad and with medical data from hospitals and other health services. However, these efforts must not divert the focus away from the urgent need to invest resources into a large Swiss population cohort and biobank, given that there is a limited return-on-investment due to the small size and the non-harmonizability of data, biological specimens, aims, and study protocols of existing Swiss cohorts.

## The Large-Scale Swiss Cohort and Biobank

### Justification

The Swiss excellence research and health systems context allows the assembly of a high-quality Swiss cohort that facilitates internationally competitive and locally relevant research as well as longitudinal public health relevant surveillance. Interesting aspects of the Swiss context for a cohort are: cultural and socio-economic diversity including related aspects such as for example food diversity, lifestyle diversity or social network diversity; geographical diversity including altitude gradient or close proximity between rural, periurban and urban space; diversity of healthcare systems across regions and cantons; high quality of clinical research to advance the phenotyping of persons; internationally outstanding basic research offering the potential to embed translational sub studies into cohort studies; highly multidisciplinary public health research community with established national collaborations fostered by the inter-university SSPH+ network in collaboration with a broad range of academic research groups and in close communication with public health practice.

In addition to serving the urgent longitudinal research and surveillance data needs of Switzerland, a population-based cohort in Switzerland can also serve as a role model and promote the setup of equivalent research infrastructures in low- and middle-income countries, where public health and research data is much needed.

**Switzerland needs its own, but internationally harmonized, large-scale cohort for several reasons:**

For the **Swiss population** to benefit in the mid- and long-term from high-quality longitudinal research that captures their context-specific chronic exposures to beneficial and/or potentially damaging broad exposome factors and their impact on health and wellbeing, while taking into account individual-specific factors (e.g., social/family/work circumstances; use and perception of environment, socio-economic situation, cultural backgrounds, genetic make-up).

For the **Swiss healthcare system and healthcare providers** to benefit from population-based long-term information to evaluate their functioning and to identify priorities for action and adaptation towards a sustainable healthcare system.

For **cantonal and federal public health authorities** to benefit from timely evidence-based longitudinal information and human biomonitoring to plan and orient public health policies and interventions and to effectively investigate and respond to technological trends, medical innovations, as well as new environmental or pathogenic health threats.

For **cantonal and federal authorities in all domains** to benefit from timely and longitudinal information on where and how to integrate health and wellbeing in their policies and planning (e.g., urban planning; sustainable food production; occupational health).

For **Swiss researchers** to be internationally competitive by 1) having access to and bringing to the international negotiating table high-quality open access longitudinal data, biological specimens and medical images on a large scale and 2) benefitting from access to international genetic and exposome big data for providing novel insight into disease processes through comparative approaches.

For **Swiss academic career promotion** in various research domains including digital and e-health to have access to rich longitudinal exposure, health and biomarker data.

For **Swiss science (science for people)** to benefit from research conducted in close collaboration with the population to assure that 1) research priorities and conduct reflect their needs and expectations and 2) that the rights and participation motivations of data, biospecimen and imaging donors are respected.

For **Swiss public-private partnerships** to benefit from ample synergistic opportunities offered by a large population data, images, and biospecimens in life and data sciences and technologies.

### Scientific Focus

To justify the high absolute costs for the large-scale Swiss Cohort and Biobank, it has to address broad and relevant questions identified by a diverse community of researchers and policymakers (see Figure 1). Listed below are important domains and a selection of research questions previously identified by a diverse group of experts (Probst-Hensch N et al. The Rational for A Swiss Citizen Study and Biobank at^21^).

#### Evidence-Base to Promote Health-in-all Policies and Primary Prevention

Health and wellbeing throughout life are influenced by a broad range of factors. To promote a healthy childhood, adolescence, adulthood and a healthy aging, the clustering of these factors and their independent and joint effects on health and wellbeing need to be studied and understood to guide policy.

Chronic diseases, including both physical and mental illnesses, and ailments evolve over the life course in part as a result of accumulation of molecular damage, tracking of behaviors, and life periods of heightened susceptibility to certain exposures. Chronic diseases and aging over the life course are influenced by a complexity of risk and protective factors, each increasing or decreasing risk by a relatively small percentage. A chronic disease or comorbidity in a single patient mostly evolves as a result of a personalized risk profile, consisting of the individuals’ genetic background and the individuals’ exposome, e.g., the entirety of external and internal factors influencing disease symptoms and incidence as well as wellbeing. The chronic long-term influences of air pollution, heat, noise, chemicals in the environment or our nutrition, socio-demographic factors, psycho-social stressors and thereby indirectly of political decisions and cultural aspects cannot be studied in the context of randomized trials or patient-based clinical research—observational long-term cohorts are the gold standard instead. Epidemiological long-term studies capturing a broad range of social determinants complemented by biomarkers (e.g., health examinations, digital biomarkers including imaging; biological material) are the only way to approach and improve understanding of the complex risk and protective patterns influencing human health. They are also the way forward to identify aging biomarkers and thereby processes of aging, for example by comparing patients with accelerated aging such as spinal cord injury or human immunodeficiency virus infection (HIV) (participants in patient cohorts) with healthy persons (participants in population cohorts) [113]. Finally, only long-term studies can assess the long-term adverse health effects of the over 40,000 chemicals marketed each year on a global scale in the context of a post-marketing vigilance system [48].

**Evidence-base to promote health-in-all policies and primary prevention**

**Population cohorts capturing a broad range of social, environmental, and economic determinants combined with biobanks/imaging and with phenotyping for preclinical and clinical endpoints are essential for understanding the following questions [114]:**

• What makes people likely to grow up healthy and well?
• What makes people resilient to adverse health circumstances?
• What makes people likely to age healthy and well?
• What makes people likely to develop a specific disease or specific comorbidities?
• Which combinations of endogenous and exogenous factors and processes predict risk of diseases and multi-morbidities (e.g., cardiovascular disease and depression) in the Swiss population best?
• What is the long-term utility of novel technologies (e.g. mobile phone apps, wearables) for promoting a sustainably healthy lifestyle and behavior?
• What are the broad effects of companion animals on healthy growing up and healthy aging? Can companion animals serve as sentinel for adverse health effects in humans?

**In the absence of large-scale cohorts, it would be difficult to assess:**

• The long-term impact of the school and family environment on lifestyle, health and wellbeing
• The long-term impact of child and adolescent preventive care and screening
• The long-term impact of sports participation and of sports programs on health and wellbeing
• The long-term health impact of chemicals and mixtures (e.g., pesticides) on human health
• The long-term impact of climate change and heat waves on human health
• The clinical utility of novel retinal or brain imaging patterns to predict the risk of mental disorders (e.g., Alzheimer’s disease) [115, 116].
• The long-term impact of gut microbiome composition on the risk of infections or non-communicable diseases and the role of diet in these associations [117].
• The mechanisms by which higher cardiorespiratory fitness longitudinally and strongly impact on mortality [118].
• The mechanisms leading to specific multi-morbidities over the lifecourse [114, 119].
• The long-term effect of diseases and their risks on the labor market and economic outcomes [120].
• The long-term cost-effectiveness of specific preventive interventions [121].
• The protective effect of air quality standards on the health of the most susceptible population groups [122].
• The characteristics of urban design that promote health and wellbeing [123].
• Chemical and occupational exposures needing regulation (e.g., pesticide mixtures) and endpoints supporting regulation (e.g., effect biomarkers) [124].
• The long-term impact of the work environment on health and wellbeing; the identification of effective stress- and suicide-prevention strategies at the workplace [125].
• The effect of high chlorine content in water on various aspects of human health?
• The effect of computer screen use on the unborn child in pregnant women; the effect of noisy jobs on the fetus in pregnant women
• The effect of living close to motorways on asthma development in children?

**Population cohort data provide the evidence-base to:**

• Improve understanding of diseases and aging over the life course from observational associations towards better mechanistic and causal understanding through refined exposure assessment and interrogation of biology and imaging
• Derive evidence-based personalized risk prediction algorithms for identifying persons at risk
• Evaluate long-term disease risk patterns from a socio-economic perspective for targeted health promotion
• Design evidence-based health-in-all policies and evaluate their impact
• Provide the basis for post-marketing chemical vigilance systems.

#### Evidence-Base to Strengthen Risk and Disease Screening

An important focus of personalized health is the identification of biomarkers and algorithms derived from biological material or imaging that signal disease risk or an already evolving disease at a very early stage. Prospectively collected functional measurements, biosamples and images, obtained before disease occurrence in the healthy state, and stored in high-quality data and biobanks can serve personalized health research into both target discovery and target testing for clinical and public health utility. Population cohorts such as the All of Us Cohort are therefore an essential part of personalized health research initiatives. In the US Precision Health Initiative more than half of the funding is invested for building a population cohort consisting of 1 Mio participants.^22^ In the first White Paper for the Swiss Personalized Health Initiative, it was accordingly acknowledged that “in a second phase, there is a need for a large healthy population-based reference cohort”. Without access to prospectively sampled biomaterial and images in healthy individuals followed-up over time, Switzerland will remain marginalized in international personalized medicine and precision health research. In the absence of cohort data with integrated prospective blood sampling we would not know for example that high blood cholesterol is an independent predictor for the risk for cardiovascular disease and we would not be able to derive a diagnostic cut-off for the initiation of blood lipid lowering therapy.

**Evidence-base to strengthen risk and disease screening**

**Population cohorts with biobanks/imaging are essential for understanding the following questions:**

• What are molecular and imaging biomarkers with utility in predicting disease risk?
• What are signs for an impending disease with utility for screening?
• What are molecular targets for novel risk and disease screening and for novel diagnostic instruments?

**In the absence of population cohorts, it would be difficult to answer the following questions:**

• Do novel biomarkers or algorithms help to predict or early detect diseases beyond current state-of-the-art strategies [126, 127]?
• What are reference ranges and diagnostic or prognostic cut off levels of novel biomarkers [126, 128]?
• Does knowledge of an elevated personal/genetic disease risk score motivate individuals to make evidence-based sustainable behavioral changes (e.g., lifestyle, screening, chemoprevention) [129]?

**Population cohort data provides the evidence-base to:**

• Assess the public health and clinical utility of novel biomarkers and disease/risk prediction algorithms evolving from personalized health research
• Assess the impact of the implementation of novel screening interventions on morbidity and mortality
• Promote the development of novel preventive and diagnostic instruments with high public health utility
• Take innovations in the domain of personalized health and biomarkers to impact and population health benefit by also assessing their acceptance

#### Evidence-Base to Strengthen the Functioning of the Swiss Healthcare System and Health Systems Research

Active disease and symptoms monitoring in the context of longitudinal studies makes it possible to measure the true prevalence of diseases and the under-diagnosis of diseases and its mid- and long-term consequences. Population cohorts can evaluate access to care and the longitudinal consequences of poor access, including the care with respect to the provision of primary prevention. Patients in population cohorts obtain health services in diverse geographical locations and health system settings so that different aspects of the Swiss healthcare system can be evaluated, as well as care provided outside of university hospitals, e.g., in smaller hospitals and in ambulatory care. These aspects are of particular relevance with regards to new medical technologies that pose novel challenges for doctors, patients, the healthcare system and society more generally, and carry the risk of increased social inequity in access. Finally, population cohorts will identify patients and incident diagnoses and can thereby feed patient registries, allow for the implementation of specific patient cohorts, and support trials-within-cohorts as long as they do not interfere with the observational nature and focus of cohorts.

**Evidence-base to strengthen the functioning of the Swiss healthcare system and health systems research**

**Population cohorts with biobanks/imaging are essential for understanding the following questions:**

• What is the true prevalence and incidence of physical and mental symptoms and diseases?
• What is the degree of under-diagnosis of diseases?
• What aspects of the care provided in peripheral facilities and in ambulatory care are efficient and of high quality–which aspects need improvement?
• What are emerging health threats at different stages of life?
• Do the Swiss healthcare systems and health professionals adequately respond to the needs of persons and patients and promote health and wellbeing in the long term?
• What are the healthcare cost and financial burden in different population subgroups as they develop diseases and age?

**In the absence of population cohorts, it would be difficult to answer the following questions:**

• What is the percentage of persons with type 2 diabetes, hypertension, or COPD who remain undetected [130, 131] and what longer-term consequences does this have?
• What is the impact of a delayed diagnosis of familial hypercholesterolemia on morbidity and mortality [132]?
• What is the proportion and long-term health state of nutrient- or vitamin-deficient persons [133]?
• What are the level and consequences of under-ascertainment of infections occurring at community level and under-diagnosing and -reporting of infections at healthcare level [134]?
• How are antibiotics prescribed and what is the long-term contribution of over-prescription to antimicrobial resistance?
• Are health interventions implemented according to recommendations (e.g., pharmacogenetics testing, clinical risk prediction rules for prevention of atherosclerotic cardiovascular diseases) and what are long-term consequences of poor adherence and poor access to medical care respecting guidelines [135]?
• What are long-term consequences of social equity in access to screening and healthcare, including personalized health interventions [136, 137]?
• What is the extent of health literacy related to health and health behavior in general, and genetics, personalized, and digital health in particular [138] and what are the long-term consequences?
• What is the long-term cost-effectiveness of (personalized) health interventions, health promotion, prevention and screening [139] in different age groups?
• Can patient counselling be individualized, based on studies of the effects of interaction between medical treatment, behavior/environment and genetics on the disease course [140] and what is the long-term benefit?

**Population cohort data provides the evidence-base to:**

• Improve the quality and cost-effectiveness of the healthcare system and its impact on population health and wellbeing
• Promote socially just use and access quality care
• Strengthen acceptability and use of novel technologies by persons and patients
• Surveilling emerging health threats, such as infections and the course of pandemics as well as climate change or the impact of public health measures by comparing pre- and post-health and wellbeing states or by investigating health trajectories

#### Swiss Bio and Swiss Imaging reference—Example for an Embedded Deep Phenotyping Sub-Study

The Swiss Tropical and Public Health Institute (Swiss TPH), in collaboration with the University of Basel and the Institute of Molecular and Clinical Ophthalmology in Basel (IOB) and under the umbrella of the Swiss School of Public Health (SSPH+) have submitted the Roadmap Infrastructure project “Imaging and–Omics Platform for Swiss Citizen Health (IOP4CH)”. The project was assigned the highest excellence level upon scientific evaluation by the Swiss National Science Foundation (SNSF) and has been shortlisted by Swiss Universities as a recommendation for a future research infrastructure. A broad national network of research institutions and public health and clinical research partners has given strong support to the planned deep phenotyping of a population cohort and biobank of at least 10,000 participants. The project evolved from the existing cohort infrastructure at Swiss TPH in Basel (COVCO-Basel cohort; epidemiologic examination center and bio-banking infrastructure consisting of −80° freezers and liquid nitrogen tanks) and plans for the implementation of MRI (1.5 and 3 T) and ophthalmology imaging infrastructures dedicated to examinations for the population cohort. This research infrastructure is scalable to other study centers in Switzerland. It is planned to develop the study protocol with a broad range of policy and research stakeholders.

This infrastructure will be applied toward longitudinal deep characterization of persons along the axis from exposome (non-genetic disease determinants, e.g., lifestyle, psychosocial, environmental risks) to mediating molecular and imaging biomarkers to aging-related co-morbidome.

**The Roadmap Research Infrastructure IOP4CH** (Imaging and Omics Platform for Swiss Citizen Health) aims at implementing the **Swiss Bio and Swiss Imaging Reference Data** based on a deeply phenotyped cohort providing rich healthy reference data, biomaterial and images accessible for national and international public, personalized and digital health research.

The foreseen roadmap infrastructure can also serve as the North-Western Switzerland research hub for the Swiss Cohort and Biobank and is scalable. Its protocols can either serve for examination of participants from other research hubs and/or can be shared and implemented in other research hubs.

The submitted roadmap infrastructure project serves as a role model for translational collaboration between different research partners and as a role model for the willingness of institutions and universities to provide relevant matching funds and infrastructures for the foreseen large-scale Swiss cohort and biobank.

### Methodological Aspects

#### Study Design

**FIGURE 2:**
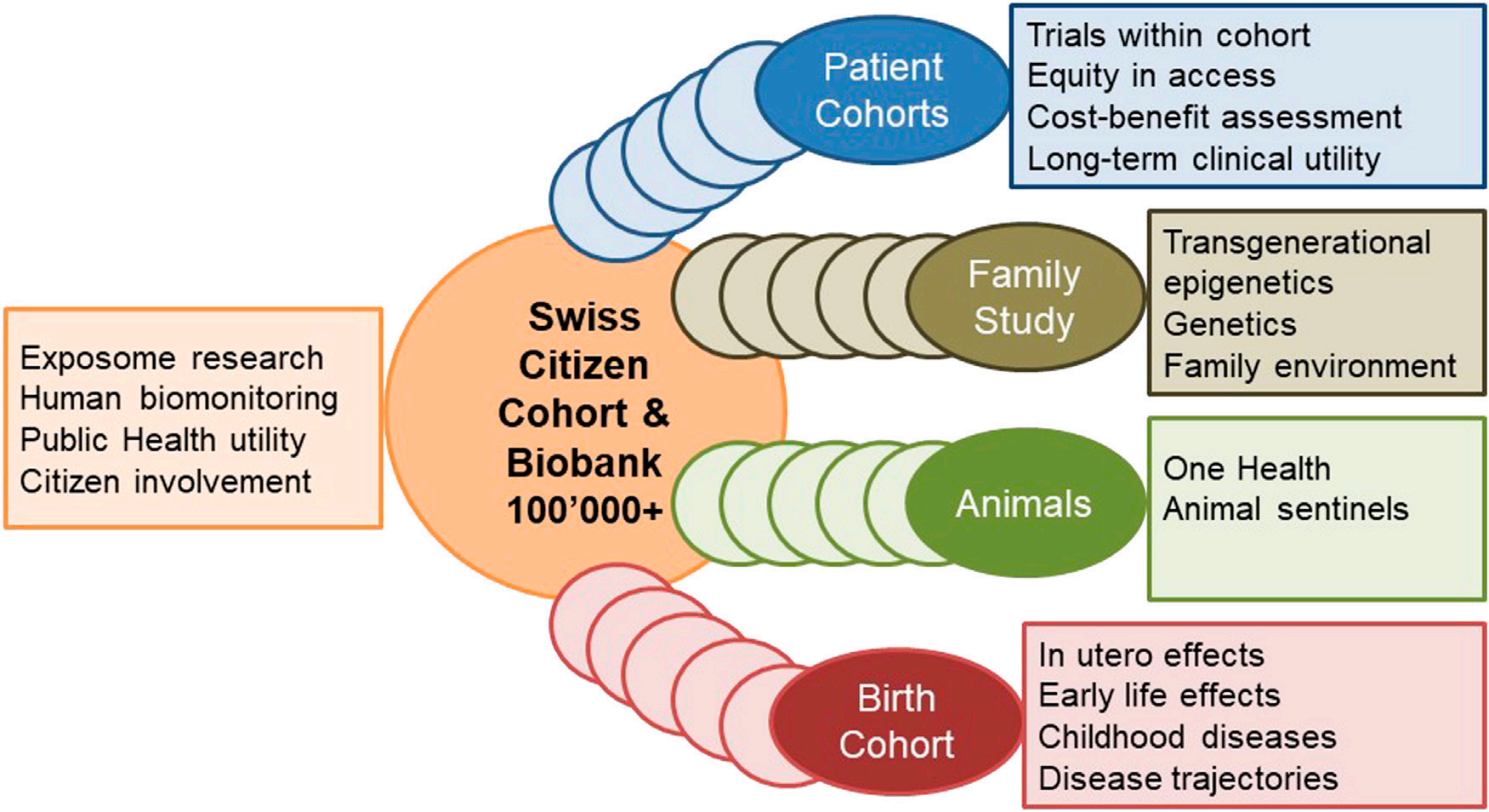
Swiss Cohort and Biobank of 100,000+ participants of all ages and options for evolving sub-cohorts.

The cohort design (see Figure 2) has to maximize the scientific and policy utility of the platform. The cohort design must be flexible enough to integrate evolving novel research questions in a timely, efficient and collaborative manner.

Cohort participants must cover the whole life-course and therefore span the range from unborn to old age. This can be achieved by age-stratified, population registry-based sampling. Age and subgroup specific study protocols can be implemented in part. As a novel approach, birth, family, and patient cohorts can be nested longitudinally into the cohort. First, identifying pregnancies at a very early stage—even at the stage of intending to become pregnant—and implementing a birth cohort for research offers the opportunity for studying early life and even pre-conception effects. The integration of family studies facilitates genetic, epigenetic and rare disease research as well as research into the role of family environments for health and wellbeing. Second, the integration of population-based patient cohorts evolving are fundamental for evaluating the performance of the healthcare system in various domains. Additionally, a subcohort of companion animal owners (dogs; cats) within the cohort would allow for novel **One Health** research approaches, such as the impact of companion animal ownership on lifestyle and wellbeing or using companion animals as sentinel for environmental exposures for chemicals. Additionally, subcohorts of specific occupations (e.g., farmers) or subcohorts of migrants can be nested into the cohort.

The design for the Swiss Cohort and Biobank can be guided by the design of the following mega cohorts established in Europe:

**NAKO** recruited a total of 200,000 residents in the age range of 20–69 years at baseline.^23^ Study participants were recruited through a network of 18 study centers, covering mainly urban and industrialized areas and some rural regions throughout Germany [103]. Each center recruited a minimum of 10,000 cohort participants.

**CONSTANCES** was designed as a randomly selected sample of French adults aged 18–69 years at study inception [104] (Constances | Améliorer la santé de demain). Between 2012 and 2019, more than 200,000 individuals were selected randomly from the database of the National Pension Insurance Fund (CNAV: Caisse Nationale d’Assurance Vieillesse).

**LifeGene** in Sweden was designed as a prospective cohort study and an infrastructure with repeated contacts of study participants approximately every 5 years. Index persons aged 18–45 years were recruited and invited to include their household members (partner and any children) [105].^24^ The household-based design gives the opportunity to involve young couples prior to and during pregnancy, allowing to study children born into cohort with complete pre-and perinatal data from both the mother and father. The target of LifeGene was initially to recruit 500,000 Swedes and follow them longitudinally for at least 20 years, but this goal has not yet been achieved and the study faced some data protection challenges.

The Canadian **CHILD Cohort Study** ^25^ is a prospective longitudinal birth cohort study, designed to collect information at time points considered critical to health and development. Participants are followed over time as they grow and develop—from mid-pregnancy into childhood and adolescence. CHILD is a research platform to understand disease development, prediction, prevention and treatment collecting biological samples, self-reported health data, indoor and outdoor environment and clinical assessments.

Avon Longitudinal Study of Parents and Children (ALSPAC^26^), is a world-leading birth cohort study in the UK that recruited more than 14,000 pregnant women between April 1991 and December 1992. Participants and their children and family members have been followed up since. The study provides the national and international community with rich data on health and disease development across generations. It informs policy and practices with evidence for a better life for future generations.

**Growing Up in Ireland** is a government-funded study of children being carried out by a consortium of researchers (Growing Up in Ireland–National Longitudinal Study of Children). It recruited 18,000 children between 9 months- and 9 years-old in 2006 and follows them up since. The primary aim of the Growing Up in Ireland study is to **inform Government policy** in relation to children, young people and families’ health and wellbeing.

The “Study on the Health of Children and Adolescents in Germany” (**KiGGS**) is conducted by the Robert Koch Institute (RKI).^27^ The first KiGGS study (baseline survey) from 2003 to 2006 provided the first comprehensive and nationally representative health data using both surveys and medical examinations, tests, and laboratory analyses to provide more valid measurements and better frequency estimates for diseases in children and adolescents. Since 2009, KiGGS cohort is being followed up and has been continued as a component of national health monitoring of both subjective and objective data.

The Adolescent Brain Cognitive Development **(ABCD) Study** **®** is the largest long-term study of brain development and child health in the United States^28^. Since 2015, the National Institutes of Health funds ABCD Research Consortium implemented by leading researchers in the field of adolescent development and neuroscience from 21 research sites across the US has invited 11,880 children ages 9–10 to join the study. Their biological and behavioral development through adolescence into young adulthood will be tracked.

#### Sample Size and Statistical Power

The Swiss population cohort should include at least 100,000 participants plus family members to be internationally competitive and sufficiently informative for the Swiss context. Stable and adequate long-term funding must be provided to secure this instrumental infrastructure for the entire recruitment and follow-up period. The latter should cover at least 20 years. The value of resampling of new participants over time will be evaluated.

The sample size need depends entirely on specific future research questions, some of which may focus on the study population as a whole, some of which may focus on subgroups only and take complex interactions into consideration. Research questions in need of a sample size larger than the sample size provided by the Swiss Cohort and Biobank will benefit from internationally harmonized study protocols and collaboration in large cohort consortia.

To obtain an estimate of the number of clinical disease outcomes evolving over time in a cohort of 200,000 citizens aged 18–69 years, CONSTANCE provided an overview of the expected number of incident major health outcomes according to different follow-up times [104]. The data demonstrates that even for non-rare endpoints such as Alzheimer’s disease very large sample sizes can often only be achieved in cohort collaboration (see Table 1).

**TABLE 1:** Expected number of incident major health outcomes according to follow-up in the CONSTANCES cohort [104].

Health endpoint	5-year follow-up	10-year follow-up	15-year follow-up
Death, all causes	6,264	15,229	27,719
Incident cases of ischemic heart disease (35–64 years)	819	1708	2630
Incident cancers	5,381	11,892	19,267
Incident cases of Alzheimer’s disease	505	1,007	4,018

#### Recruitment Approaches—Minimizing Loss-to-Follow-Up—Maintaining Sample Size

Intensive efforts must be made to minimize attrition of the cohort due to loss-to-follow-up. Experience from running large cohorts will need to be respected and validated in the Swiss context. While the minimization of loss-to-follow-up is the highest priority in a cohort study, its recruitment should still also aim at representativeness in the Swiss context, given that the cohort should also aim at providing policy relevant surveillance data on the longitudinal distribution of health trajectories, healthcare services provided and exposures and their fluctuation across different population strata. For strengthening surveillance needs, it is also important to harmonize instruments between the cohort and relevant cross-sectional surveys.

Stratified sampling of participants from population-based registries is the gold standard that was successfully implemented by Swiss population cohorts such as SAPALDIA [62, 141], CoLaus [142], and SOPHYA–the population-based national study on physical activity in Swiss children [143]—, Bus Santé [144], and most recently by the Corona Immunitas program [41]. This sampling approach was also implemented in the pilot phase for the population cohort (Swiss Health Study of FOPH). Yet, additional sampling sources and approaches need to be considered for reaching out to and involving underrepresented population groups, e.g., persons with a migration background.

Recruitment into mega cohorts in different Western European countries also mostly followed the approach of random sampling from population registries.

For the sampling for NAKO random samples of the defined source population for each study center were drawn in the respective city or community registry, stratified by 5-year age groups and gender. The age criterion was 20–69 years at the time of sampling. Age groups aged 40–69 years were oversampled, giving the lower age groups 20–39 years a slightly lower weight than higher ages [103].

The sampling for CONSTANCES was done within the database of the National Pension Insurance Fund (CNAV: Caisse Nationale d’Assurance Vieillesse) [145]. Almost none of the people included in CONSTANCES will be permanently lost to follow-up, since the participants are followed passively through the national administrative databases.

For the sampling for LifeGene, index persons (aged 18–45 years) were randomly sampled from the general population, with oversampling of twins from the Swedish twin registry. Participants were invited to include their household members (partner and any children).

In order to maintain a sufficiently sized cohort, different approaches can be considered. First, new representative samples can be invited into the cohort to replace participants lost-to-follow-up for different reasons. Of more benefit scientifically is an approach that invites participant’s family or social network members of different age groups and generations into the cohort, as this additionally allows studying the distribution of health and its determinants within social networks, that are in part genetically related.

#### Linkage to Health and Other Relevant Outcomes

An effort must be made to link the participants in the future Swiss Cohort and Biobank with registry-based information including the Swiss National Cohort and its associated data structures to evaluate and correct for selection bias and to improve generalizability of future results.

In addition, the linking of the Swiss Cohort and Biobank with medical records as made available by the Swiss Personalized Health Network will be important for research purposes and the identification of health endpoints developing in participants. Linking the cohort participants to electronic medical records with explicit consent of participants will be a very important goal. In the meantime, given the current lack of Swiss-wide registries in various domain and given that the use of the electronic patient file for research purposes is not yet assured, it will be crucial to obtain proxy information from cohort participants, in that they allow persons in their social and healthcare provider network to be approach for finding out about future diagnoses and whereabouts of participants in the case of situations, where the participants move out of the country, no longer live at home, develop a disease where they can no longer be interviewed, or die.

#### Participant’s and People’s Engagement

Population cohorts have the advantage of being in direct and close contact with various subgroups of the population. Ideally, persons perceive the cohort as their “own” study, serving their needs. Active exchange with participants in longitudinal studies assures optimal participation in the cohort and its follow-up assessments [51, 94].

Participatory community research enables understanding and acceptance of the expectations and research of healthy persons and specific patient groups. Involvement of different citizen subgroups in prioritizing and shaping research priorities and approaches in a large scale cohort has the potential to achieve a higher acceptance and impact of cohort findings in the future. Community-based participatory research ideally involves community members, policy stakeholders and researchers jointly in all steps of the research process to assure that expertise, decision taking and responsibilities are shared. For example, a list of the following eight principles or characteristics of CBPR has been recently identified [146]: “1) recognizing community as a unit of identity; 2) building on strengths and resources within the community; 3) facilitating collaborative partnerships in all phases of the research; 4) integrating knowledge and action for mutual benefit of all partners; 5) promoting a co-learning and empowering process that attends to social inequalities; 6) involving a cyclical and iterative process; 7) addressing health from both positive and ecological perspectives and 8) disseminating findings and knowledge gained to all partners.”

Cohorts that are run in partnership with participants and with experts in life sciences and public health, social sciences, ethics and law, as well as communication and social marketing have the opportunity to enhance understanding of mutual expectations and needs. In particular, they can promote the understanding of citizens of the value of participation in research and of donating data, biospecimens and time to improve population health. Cohort participants can become important actors in communicating these highly relevant societal needs. Furthermore, empowering study participants with data and information to improve and promote their own health through evidence-based decision-making from a sufficiently powered cohort has the potential to make a significant direct contribution to improving people health. Finally, cohorts paralleled by professional communication strategies in the context of effective information channels help educate persons, patients, and health professionals about novel findings and technologies. Thus, a population cohort also serves the clinical research platform recently established by the Swiss Academy of Medical Sciences SAMW as a sounding board and communication target.

#### Data and Health Examinations

A broad stakeholder involvement process, organized in dedicated working groups, is needed in the detailed planning of the final Swiss Cohort and Biobank study protocol, both for the shared basic protocol (age-independent and age-specific basic protocols) and for sub-group specific additional protocols. As far as possible, a Swiss population cohort must thereby adapt its in-person and digital data collection, interview, health examination and phenotyping, bio sampling, and imaging protocols to internationally harmonized protocols developed by and adopted by mega cohorts of children and adults abroad. This will assure that the Swiss Cohort and Biobank in the future can participate in international research programs and consortia for investigating public health and digital and personalized health research questions with sufficient statistical power. But it is also essential that study protocols are harmonized with existing public health surveys and with cohorts in Switzerland wherever possible. Furthermore, the study protocol and data to be obtained must meet the needs and expectations of the policy stakeholders, research stakeholders, and private stakeholders to assure their investments, both in-kind and cash into this longitudinal research and surveillance platform.

These harmonization principles were already reflected in the pilot phase for the Swiss Health Study, which developed its procedures based on already existing protocols of different studies, most importantly the SAPALDIA study, but also HBM4EU, MenuCH, BusSanté, NAKO, GerES, and CONSTANCES. In the Swiss Health Study pilot phase, a biological sampling protocol has been developed and implemented under the guidance of the Swiss Biobanking Platform and in collaboration with numerous laboratory science experts, which can guide sample collection and processing. The SPHN Driver Project SACR, which brings together three population cohorts for a joint brain MRI protocol can provide guidance in implementing MRI imaging protocols and storing and annotating imaging data. Yet, an additional scanning of broader and to be prioritized protocol aspects is needed, in particular also with regard to children’s cohorts.

Like any other cohort, large scale cohorts have to adjust their examination program and, thus, the depth of phenotyping, to the priority study aims, logistics and funding [147]. Given the high absolute costs for implementing and running a large cohort, it ideally serves many research domains and topics to justify the costs and to increase their acceptance and willingness to invest by the research, policy, societal and private community. The long-term use of these data for research and surveillance purposes must be envisioned and the study design must be sufficiently flexible to integrate newly arising topics. “General-purpose cohorts” in epidemiology and public health are therefore designed to cover a broad scope of determinants and outcomes, in order to answer several research questions, including those not defined at study inception. But the broader the focus of a study, the less detailed are the exposures and phenotypes assessed and the participant time allocated to examinations of one specific phenotype [147]. There should thus be an *a priori* decision about priority research areas and length of participant examinations. The protocol must consist of core protocols (age-independent and age-specific) applied to all study participants in principle, and of subgroup specific protocols.

The UK Biobank study for example used an examination program that lasted about 90 min for the initial 500,000 participants and assessed many phenotypes by applying very short or screening versions of established scales to meet the limited examination time [147]. In-depth phenotyping was integrated into the cohort for subgroups and over time.

The overall goal of NAKO [103, 148] is to assess risk factors and determinants of major (‘frequent and/or severe’) diseases on a population level, operationalized in eight major disease groups. Given the broad research scope participants are examined at two levels: Level 1 is the standard program that lasted about 4 hours at each study center and Level 2 is an extended program lasting in total about 6 hours additionally applied to a randomly chosen sub-set of participants. The investigators allocated similar amounts of examination and interview time to each of eight research areas in the standard program. To increase the depth of phenotyping additional examinations for most, but not all, research areas were added in the extended program. To allow an efficient cost benefit ratio it was decided that 80% of the participants receive the standard program and 20% the standard plus the extended program. This approach has the advantage of recruiting a large sample size, examined for a broad range of phenotypes and research areas while still providing an in-depth phenotyping for a random 20% of study participants.

Thus, distribution of study instruments between the base protocol applied to all participants versus additional protocol applied in specific subgroups as well as re-application of study instruments during follow-up needs careful evaluation.

Large cohorts such as the NAKO in Germany, CONSTANCES in France, and LifeGene in Sweden as well as the children and birth cohorts listed earlier can guide the data and examination protocols for the planned Swiss population cohort of children and adults of different ages as they share the broad focus of the established cohort.

**NAKO** has four main research objectives, 1) the identification of pathways from lifestyle and environmental risk factors to major diseases; 2) the analysis of geographic and socio-economic disparities in health risks; 3) the improvement of prediction models for those at increased risk of specific diseases; and 4) the identification of bio- and imaging-markers for subclinical disease [103]. The scope on frequent and/or severe diseases includes eight disorder groups, cardio-vascular disorders, metabolic and lung diseases, cancer, musculoskeletal diseases, psychiatric and neurologic disorders and syndromes and infectious diseases. In 5 of the 18 study centers dedicated 3.0 T S MRI scanners were set up for a whole-body MRI of L2 study participants [148]. The NAKO examination battery also includes 3D Full Body Surface Scans in several study centers. With 3D full body scans the body shape *in toto* can be followed over time with high precision and accuracy, and approximately 140 anthropometric measures are taken within 10 s [149].

Baseline examinations started in early summer 2014 and ended in summer 2019. About 2.5 years after baseline each participant received a written questionnaire to report the onset of new diseases since the first examination. In 2019, the first follow-up examination started for which all participants are re-invited, on average 5 years after baseline. In addition, a linkage with their health insurance data is pending for each participant consenting to this procedure.

**CONSTANCES** objectives overlap with those of NAKO, but lack an imaging protocol [145]. It has a broad focus on occupational and social factors, on chronic diseases and on aging [104]. At inclusion, the selected participants were asked to complete questionnaires and were invited to attend one of the 24 participating Health Prevention Centers located in 21 cities throughout metropolitan France for a comprehensive health examination including biometry (weight, height, waist and hip circumference), blood pressure, electrocardiogram, vision, audition, and spirometry. An active follow-up included a yearly postal or web-based self-administered questionnaire, and a complete 4-year follow-up including a health examination. Moreover, data are regularly extracted from the French administrative and health national databases, including hospital discharge summaries, visits to health professionals, medication and other prescriptions, severe chronic diseases, sick leaves, handicaps, disabilities and injuries, cause of death, as well as social and demographic characteristics, socioeconomic and employment status. Extensive procedures have been developed to use the national healthcare databases to allow identification and validation of diseases over the follow-up. Blood and urine samples were collected for biobanking as well as for measuring biological parameters related to liver or renal functions, dyslipidemia, glucose metabolism, whole blood cell counts and to communicable diseases such as Hepatitis B and C, HIV, sexual transmitted diseases. Specimens are collected according to a standardized protocol, identical in all recruitment centers. All operations relating to bio-banking have been entrusted by Inserm to the Integrated Biobank of Luxembourg (IBBL). A quality management system has been put in place. Particular attention has been paid to the traceability of all operations. The nature of the biological samples stored has been deliberately limited due to the economic and organizational constraints of the inclusion centers. Some research works may require specific collection conditions, and can be developed on request for a limited number of subjects and in specially trained centers. Constance explicitly offers to the research community the option of nesting specific research protocols into the cohort.

In **LifeGene** in Sweden, a comprehensive questionnaire addressing cutting-edge research questions was administered through the web with short follow-ups annually. Biosamples and physical measurements were also collected at baseline, and are planned to be re-administered every 5 years thereafter. Event-based sampling is a key feature of LifeGene. It will include reporting of Influenza-like symptoms, pregnancies, and injuries [105].

There are three main categories of study participants entering the web portal: adults aged 18–45 years (index persons) or older, children invited by index persons and the parents to these children. The adult library holds approximately 1,350 questions and the child/parent library approximately 1,150 questions [105].

The questions are available to the study participants through a web portal, showing a circular clock-like menu with questionnaire themes on the dial. Nine themes are shown to adults: Lifestyle, Self-care, Woman’s health, Living habits, Health history, Asthma and allergy, Injuries, Mental health and Sociodemography, and between four to nine themes to the partners and children. Parents answer for their children aged 0–14 years and children answer for themselves from 11 years and up, which means that there are parallel questions to children and parents between the ages of 11 and 14 years [105].

**FIGURE 3:**
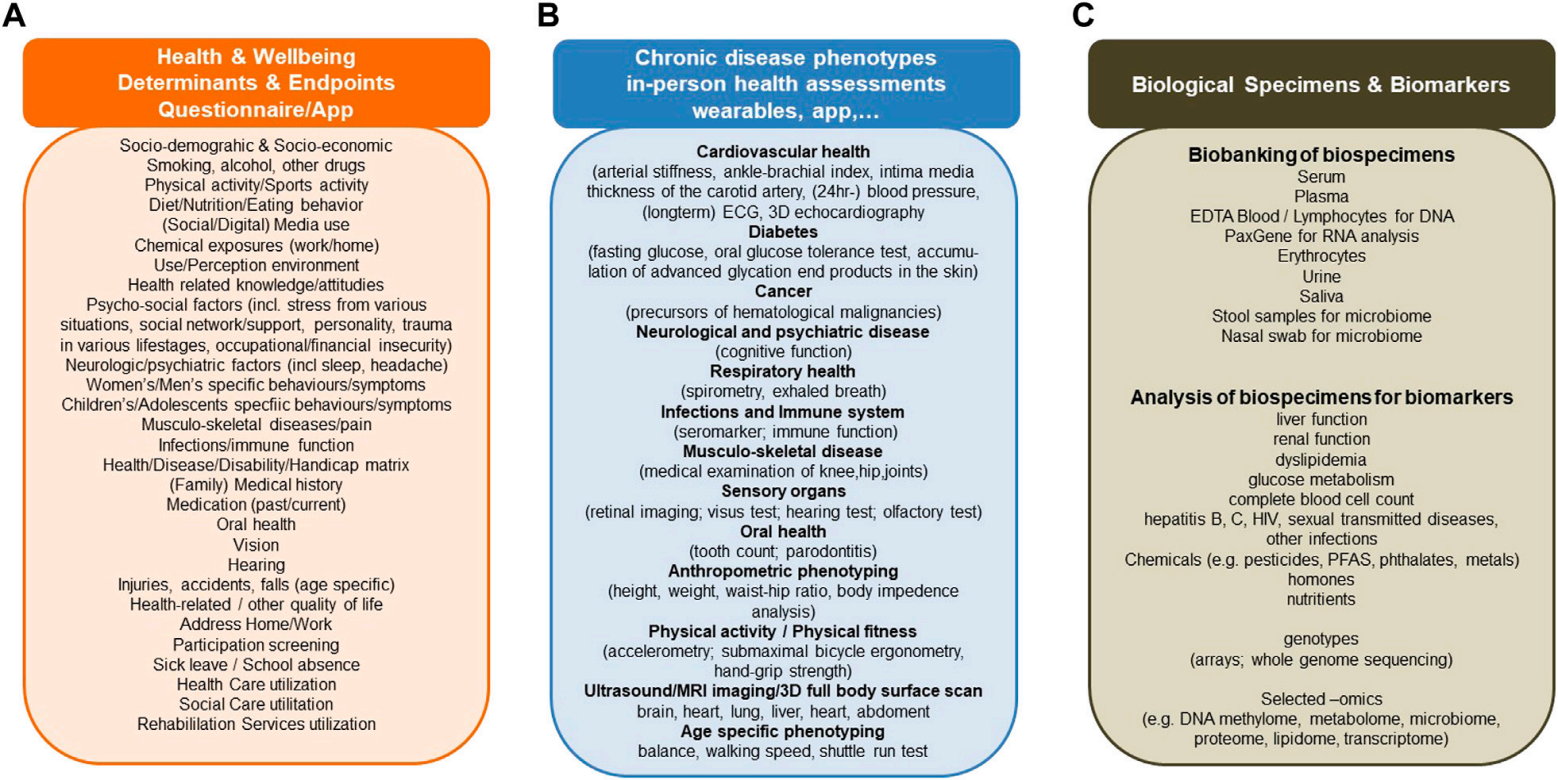
Determinants and endpoint data **(A)**, health and phenotype assessments **(B)** and biological specimens and biomarker domains **(C)** to be considered by the Swiss Cohort and Biobank, based on existing cohort study protocols [103–105].

An overview of data to be collected in the context of the future Swiss Cohort and Biobank is included in Figure 3 below and takes into consideration data collection of NAKO.

#### Data Management and Access

The pilot phase for the Swiss Health Study in collaboration with Unisanté and Swiss TPH has developed a participant and data capturing and management system that was later also adopted by the Corona Immunitas program. It uses RedCap as a secure web application for building and managing online surveys and databases. The data capturing and management system is paralleled by a biospecimen management instrument and needs to be expanded to capturing and processing imaging data.

Data, images, and biospecimens obtained should be described, coded, and searchable with the help of meta-data catalogues from Maelstrom Research. This standardized study description model with variable classification ontology is currently applied in SPHN to Swiss cohort and registry data and supports the findability of data in general. Descriptions of biospecimen collection and processing must closely follow the guidelines developed by the Swiss Biobanking Platform and include their expertise.

Research data, biological samples, images, biomarkers, and raw data from health assessments must be accessible for processing, molecular analysis and statistical or artificial intelligence (AI) analyses to the research community in the context of structured request and approval procedures as currently being developed and implemented by SPHN. Data hosting and data transfer as well as data analysis must benefit from the infrastructure and processes currently developed within the SPHN network and its Data Coordination Center (DCC).

Scientific ownership and intellectual property guidelines are needed and their development may benefit from the successful SSPH+/Corona Immunitas model. Pricing for access to biospecimens, images and research data will be different for public organizations and academic research.

SPHN has funded several projects over the past years to support the development streams for different types of data. These projects are committed to Open Research Data principles and best practices. It will be important that the Swiss Cohort and Biobank closely aligns with SPHN on all relevant topics of data management, data access, data interoperability, data security, etc. The Swiss Cohort and Biobank can thereby benefit from investments into SPHN infrastructures and processes and from the expertise developed during this process. Comparability of data, safety and interoperability standards between the Swiss Cohort and Biobank and SPHN standards will be important to make public health data and clinical data comparable.

#### Ethical and Legal Considerations

The Swiss Cohort and Biobank is ideally funded as a research infrastructure. This will assure the needed flexibility in data collection and design and follow the principles of cohorts as research infrastructures in other countries.

The consent obtained by cohort participants must be broad and offer participants to consent separately to different aspects, including the sharing of research data with partners abroad and with private partners to support international big data collaboration and public-private partnership collaboration. Participants must be transparently informed about the role of private funders and they must have the opportunity to not consent to the development of commercial products based on their data.

The pilot phase of the Swiss Health Study in collaboration with Swiss TPH and Unisanté has developed an ethics protocol and study consent for a population cohort and associated biobank that can guide the ethics protocol for the future large-scale cohort. Adaptations are needed to adequately address ethics and consent in minors.

The ethical and legal considerations will also need to provide clear guidance on dealing with incidental findings, in particular as they relate to genetic data and imaging data. These aspects need to be developed in close collaboration between experts from ethical, legal, clinical and public health domains.

### Governance

#### Swiss Cohort and Biobank: A Public Health Task

Developing and implementing a population cohort requires epidemiological training and multidisciplinary expertise. It is a core task and competence of public health researchers. Public health researchers are experienced in dealing with the complexity and multidimensionality of people’s health and in tackling public health challenges through multidisciplinary collaboration with sound evidence-based approaches. The Swiss national umbrella network of SSPH+ assembles more than 250 faculty members from over 70 academic institutes affiliated with the SSPH+ Foundation universities. This virtual faculty unites more than 40 scientific disciplines of relevance for public health.

The Swiss public health research community has been the driving force for the planning of a Swiss Cohort and Biobank in the past decade. A paper published online in 2018 by Probst-Hensch and 70+ Swiss scientists underscored “the importance of complementing routinely collected health data including e-health data by population-based data and biobank.”

The study design for the pilot phase of the Swiss Health Study was developed under the scientific lead of Swiss TPH in close academic and policy collaboration with Unisanté, the Swiss Biobanking Platform and the Federal Office of Public Health. The pilot phase was conducted by public health research institutions that applied a harmonized study protocol in two centers covering two language regions. Bio-sampling protocols were developed to fulfill international guidelines in collaboration with the Swiss Biobanking Platform funded by the SNSF.

The Corona Immunitas program coordinated under the umbrella of SSPH + demonstrated the feasibility of an efficient and collaborative setup of a national seroprevalence cohort during the pandemic by public health research institutes across the country.

#### Planning the Swiss Cohort and Biobank: A Broad Stakeholder Task

**FIGURE 4:**
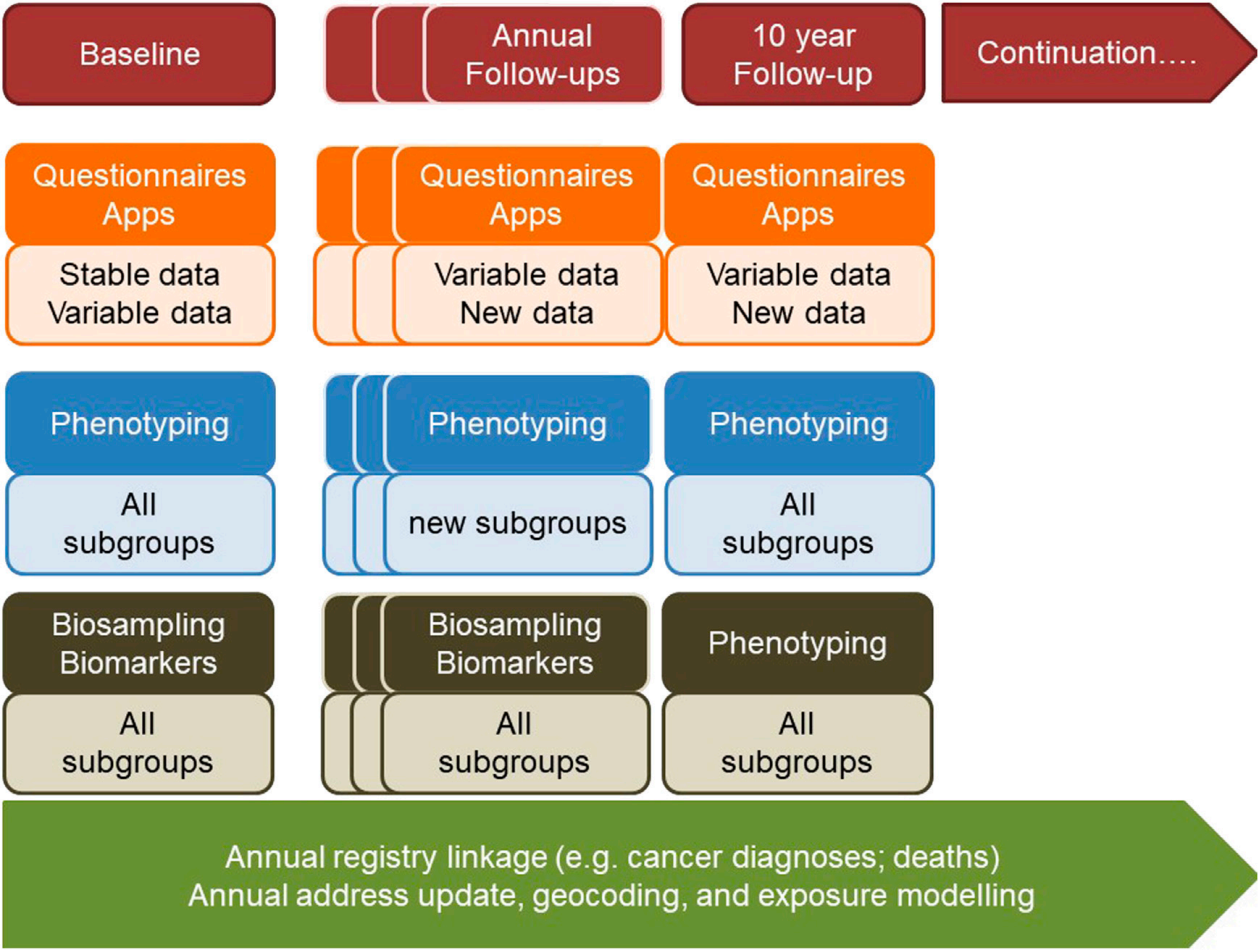
Framework for distribution of study instruments between sub-groups and over follow-up of cohort.

As the Swiss Cohort and Biobank has to satisfy broad science and surveillance needs for data, biological specimens and images, the study has to be planned in the preparation phase in collaboration with a broad range of science and policy stakeholders as well as funders (see Figure 4). What is measured and what is asked in all participants, in participants of certain age, or in other subgroups and at which follow-up time point needs to be defined and prioritized in science-and-policy collaboration and in the context of dedicated working groups involving relevant experts. The study protocol must thereby also reflect the expectations of different funders.

**The public health research community has the competencies, experience and the will to jointly develop and implement a Swiss large-scale population cohort in close translational partnership with other scientists and with policymakers.**

#### Main Guiding Principles for the Development of the Swiss Cohort and Biobank

• Pursuit of the best collaborative, competitive, and high quality science in the most ethical, sustainable and efficient manner possible
• Recognition of contributions, needs, and rights of individual scientific researchers. Contributing investigators and institutions should not be reduced to data collection, but be granted the opportunity to pursue own research questions. For that purpose, local institutions should be offered the opportunity to collect additional data and bio specimens for their own specific research interests—while they must adhere and contribute to the core protocols
• Recognition of and respect for contributions, rights, needs, and privacy of study participants in close science-population collaboration
• Funding as a research infrastructure to assure the flexibility and timeliness in data collection and design needed for research and policy - following the principles of cohorts as research infrastructures in other countries
• Local and regional public health research institutes represent the regional hubs. The regional hubs with teams reach out to collect people data. This decentralized model has been proven to be very efficient in the 31 years of SAPALDIA experience and also in large cohorts abroad. Moreover, the 2020 launch and maintenance of the SSPH+ Corona Immunitas program with its more than 50,000 participants of 40 collaborative projects would not have been possible without the local hubs, expertise and initiative to measure the development of immunity and to investigate the dynamics and consequences of the pandemic.
• The basic study protocol must be harmonized internationally, with relevant surveys and cohorts in Switzerland, and across the Swiss study centers. Close collaboration and partnership between public health, different research and policy stakeholders in Switzerland and abroad is essential for developing the study design, for obtaining funding and for assuring that the cohort meets the needs and expectations of funders. The study protocol must be developed within working groups for specific diseases and phenotyping, specific exposures, specific biomarkers, as well as specific infrastructural domains (e.g., biostatistics, data management, IT, biobank). The respective working groups must integrate epidemiological, basic, and clinical research and policy perspectives. Existing national and international study protocols and infrastructures must be respected. Policy needs need to be taken into consideration. Based on the combined recommendations of all groups, study design, recruitment and assessment schemes and protocols should be established. Priorities in policy and research data needs must be reflected in deciding on data to be collected on all study participants versus data to be collected in subgroups.
• Linkage of the large-scale cohort participants to disease and mortality registries, to diagnosis data, and to relevant administrative data must be facilitated to avoid bias due to loss-to-follow-up or outcome misclassification and to improve cost-effectiveness of endpoint identification. A unique citizen identifier allowing for efficient data linkage in public health and clinical research will be promoted. Lessons-learnt such as the challenges faced by the LifeGene Study in Sweden must be taken into consideration
• Application of information and consent procedures supporting linkage to relevant disease and administrative registries including electronic patient records, and supporting national as well as global public-private partnership
• Application and integration of current and emerging technologies, infrastructures, and protocols for data and biomarker collection, processing and storage in a secure and privacy-protecting manner. A central data hub for science in line with national and international regulations on data protection and data sharing should be foreseen. The cohort needs to establish or get access to centralized regional lab structures including examination labs, a biobank, data platforms and apps to fully integrate current and emerging technologies and–omics technics. A close collaboration with the SPHN and the SAMW Clinical Research Initiative is needed in designing the platform to maximize synergies in overlapping infrastructure domains, regulations and organizations. A close collaboration with Swiss Biobanking Platform and its partners is needed to align biological sampling with international standards
• Adoption of FAIR principles for the study instruments and collected data and metadata
• To reach its scientific goals and to successfully run with its participants, a transparent agile governance structure is needed

#### Proposed Governance

**FIGURE 5:**
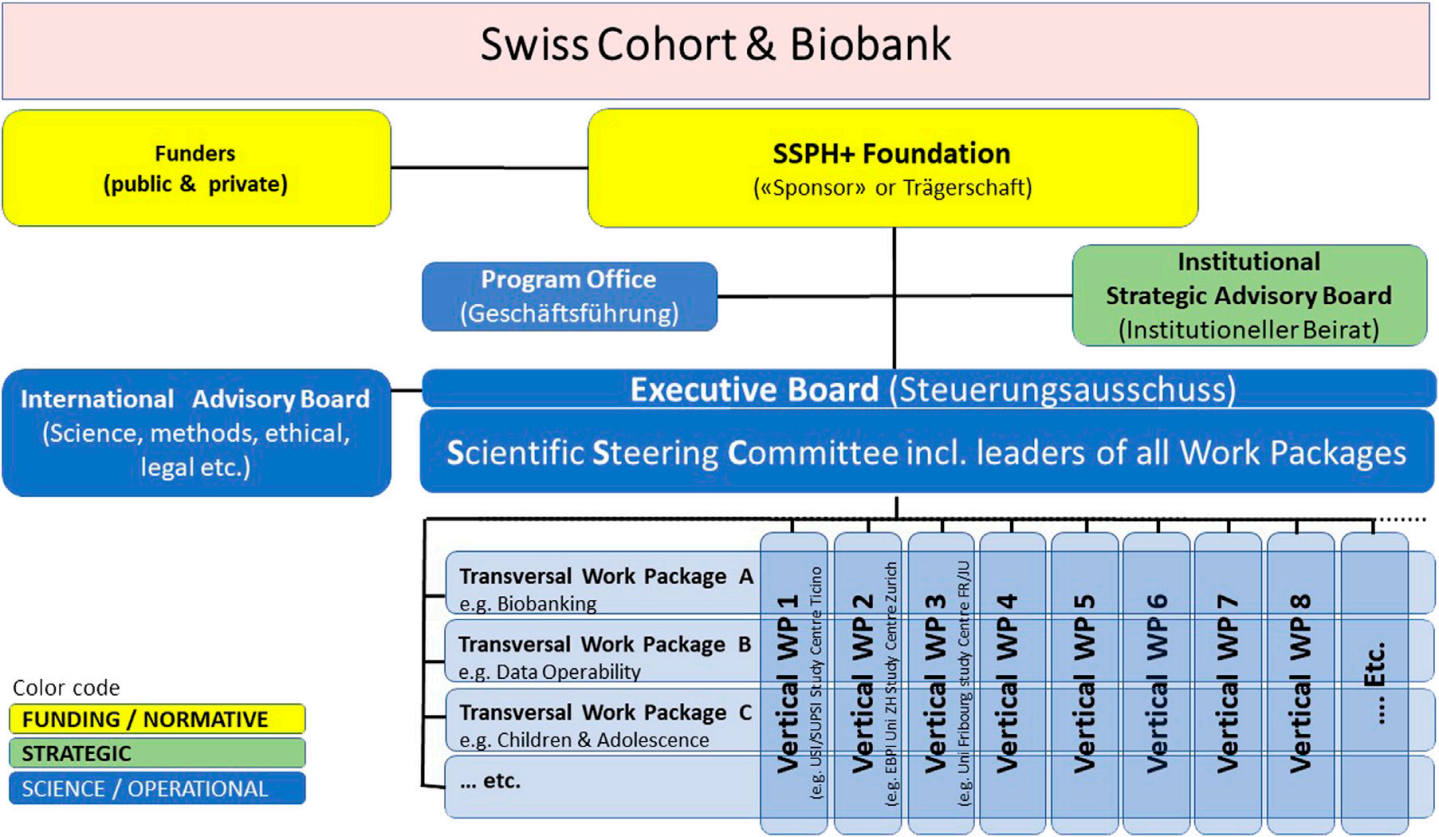
Proposed governance structure for the organization and responsibilities of the Swiss Cohort and Biobank, led by the public health sciences community targeting though questions an priorities set and monitored by public authorities and constituencies represented in the Strategic Advisory Board (details see text).

The proposed organization is visualized in Figure 5 and has been respected by the Roadmap Research Infrastructure project IOP4CH (see above). The following two principles build the underlying rationale for the proposed governance:

1) Trust: A population cohort is fundamentally dependent on the trust of people into the responsible leaders of such long-term projects. A prime underlying principle to build trust and keep participation high over time is a transparent and clear organizational structure that omits conflicts of interest and redundancies. Most importantly, funders—both public and private—must be separate from the academic community in charge of conducting the science and exploiting the data obtained. Funders and/or the public (e.g., policymakers) may co-define objectives or questions to address. It is the responsibility of researchers and population scientists, though, to define, develop and implement the design, research methods, infrastructures, teams or tools to collect, analyze and publish data, and to achieve the objectives in a scientifically sound manner. All aspects of the study, from design to data analysis, must include relevant expertise to assure high quality data and data interpretation. Open Research Data principles must be followed and re-use of data by third parties be promoted. Study participants must have a transparent understanding of the study governance and the roles of different stakeholders involved in setting research priorities, in designing the study as well as in analyzing and disseminating the data. In the context of study information, consent procedures, and different communication instruments, the trust of participants must be gained and maintained. Although structural conditions differ across national cohorts such as, e.g., the UK Biobank, the German National Cohort NAKO, the French large cohorts CONSTANCES, all those research infrastructures comply with this paradigm.
2) Governance follows function: the governance needs to ultimately fit the functions needed to comply with the purpose of the project. Thus, governance structure may change over time as new functions emerge or initially relevant ones become obsolete. Though details will depend on the objectives of the Swiss Cohort and Biobank, some functions need a transversal cross-cutting organization whereas others require vertical pillars (see details below).

The proposed organization takes into account lessons learnt and the experience of other large national population cohorts, namely the German National Cohort NAKO, the French cohort CONSTANCES, the UK Biobank with its 500,000 participants and the Vorarlberg Cohort (180,000). The proposal distinguishes three governance domains, namely; Normative Bodies (including the funding), Strategic Bodies and Operational Bodies, as outlined below.

##### Funding and Normative Bodies

Three pillars will guarantee funding: 1) The infrastructure including core staff of the cohort platform needs to be sustainably and primarily publicly funded. As a large-scale long-term research infrastructure, the cohort is fully in line with the Swiss research strategy outlined in the SERI Roadmap 2023. The conditions for contribution by private funders need to be clearly defined, but be an important option. 2) Coordinated by the Executive Board (see below), the SSPH + Foundation and the scientists will compete locally, nationally and internationally for the acquisition of public and private funds for research projects making use of the infrastructure embedded in the cohort. This may be funds to analyze existing data or as well to collect additional information from the cohort or some subsamples of the cohort or from biosamples and images. Open access data and protocols will stimulate these additional funds brought into the cohort. A separate pricing policy will be developed for non-academic and private parties with an interest in data, biospecimens or images. 3) The academic institutions in the SSPH+ and other research networks provide substantial in-kind contributions to guarantee the success of this national program (university infrastructures, academic teams etc.). Though impossible to quantify, this asset of the Swiss academic landscape is highly relevant for a population cohort. Given the funding structure of SSPH+ with a core budget being covered by the SSPH + Foundation universities, part of the in-kind contribution is provided through this funding path. Additional in-kind and cash funds from a broader set of research institutions will need to contribute and will assure that the highest quality science in different domains can be achieved.

To define clear responsibilities and accountability and to foster coordination and standardization, it is essential to define the prime legal constituency that links the funding bodies with the research community. In the absence of a pre-existing national academic institution with the proper national characteristics, the German NAKO created a “Association/Verein”. The pre-existence of the SSPH+ Foundation provides an ideal setting for this national role. With Corona Immunitas SSPH + has indeed demonstrated the power of this highly successful model. The Roadmap Infrastructure project IOP4CH would be running under the umbrella of SSPH+ also.

As the «Träger» (i.e., the “sponsor”), SSPH+ takes on the overall responsibility for the cohort, including the legal responsibility for the financing, contracting, initiation and management of the program. It mandates the tasks and duties of the Program Office (Geschäftsführung). The responsibilities include the contracting of funders to pool resources and the agreements with the receiving research institutions which guarantee smooth, scientifically sound and sustainable implementation of the national multidisciplinary and multi-centre research infrastructure, led by its operational bodies (see below).

As a Foundation, SSPH+ is independent of the research institutes that ultimately lead and conduct the project. This institutional separation guarantees unbiased and fair adoption of tasks and roles and the monitoring of the program quality. The SSPH+ Directorate office is a lean administrative structure set up to coordinate and facilitate the national cooperation in public health sciences and training. Thus, SSPH+ mandates the lead and operation of the research to its partner institutions. As a not-for-profit academic Foundation, SSPH+ also profits from its Foundation Board which oversees and approves the financial commitments of SSPH+.

##### Strategic Bodies

The Institutional Strategic Advisory Board ISAB is the prime oversight board advising on the general strategic directions of the cohort. ISAB members bring broad expertise from public federal and cantonal constituencies (e.g., Federal Office of Public Health (FOPH), Federal Office for the Environment (FOEN), Cantonal Health Director’s Conference (GDK), Swiss Health Observatory (OBSAN), States Secretariat for Economic Affairs (SECO), Federal Food Safety and Veterinary Office (BLV), Swiss Council for Accident Prevention (BFU), academic institutions or networks such as swissuniversities, ETHZ, EPFL, the academies (a+), the Swiss Personalized Health Network (SPHN), the Swiss Biobanking Platform (SBP), professional associations (in particular Public Health Schweiz and other national health-oriented organizations like FMH and patient organizations) as well as charitable or private public health and cohort/biobanking relevant industries (e.g., insurers, pharmaceutical industry, biobanking industries, food industry, wearable/sensory industry). SSPH+ assembles the ISAB once a year as an exchange platform between at least the Executive Board and ISAB. ISAB members get regularly informed about the progress and results of the Swiss population cohort. Authorities and the research community may discuss the final number and list of ISAB members. Given the multidimensional content of the population cohort the ISAB may benefit from the inclusion of up to 25 members. Those could be approved for 4-year terms by the Federal Department with the final lead of the population cohort. In preparation of the Swiss Cohort and Biobanking design and content, an intensive collaboration process between working groups and the ISAB under the supervision of the executive board will take place to assure that ISAB member needs and expectations are adequately reflected.

##### Operational Research Bodies

The Executive Board (Ausschuss) (EB) is the leading scientific authority of the cohort research program (Steuerungsausschuss). Its Chair acts as the “CEO” of the cohort. The EB is the leading body and representative of the national Scientific Steering Committee. It leads all decisions to guarantee the successful conduct of the research program, in line with the general objectives and strategic goals, the Terms of Reference agreed with SSPH+ which corresponds to the contracts signed with funding bodies, fully supported by the Program Office. SSPH+ constitutes the EB based on proposals of the Steering Committee in a way to optimize the representation of scientific expertise but also all regional hubs. Based on proposals of SSPH+ the Steering Committee elects the Chair of the EB for periods of at least 2 years. The EB may opt for the nomination of two Deputy Chairs. Advised by the Steering Committee members, the EB defines the Work Package structure of the population cohort.

The Scientific Steering Committee (SSC) is the assembly of all relevant scientific and operational research domains. Thus, at least all chairs of vertical and transversal Work Packages join the SSC (for examples see below). Others may become members if the Executive Board considers the expertise necessary for the successful management of the research program. Steering Committee plenary meetings will be organized at least annually by the Program Office. Ad hoc events supplement the decision-making process of the Executive Board.

The SSC assembles academic leaders in public health sciences, affiliated with public research institutions. They not only bring long-standing experience in the implementation and running of longitudinal studies conducted with the population, but are trained in research ethics and legal matters to guarantee compliance with all rules and regulations. Those play an important role in population-based research where the protection of data and the privacy of participants is key. Through established procedures, coordinated by the Program Office, the research team will derive secure anonymous data to be made available to the national and international science community for the advancement of research and knowledge at large.

As the central operational management unit, the Program Office (PO) guarantees the administration and coordination of all procedures necessary for the implementation of sound research activities by the academic partners, in line with the terms defined by SSPH+ as the «Trägerschaft» (i.e., the “sponsorship”). It distributes the funds, organizes all contracts, boards and committees and the internal and external program communication. It is a prime support pillar for the Chair/CEO, the Executive Board and the Scientific Steering Committee of the cohort. As the key operational unit for all centralized matters of the cohort it closely collaborates with the local operational units in charge of work packages and other decentralized activities. This includes in particular responsibilities for the national coordination of the strategies to promote the cohort to the public to reach high compliance and participation. The PO prepares also the media communication and information material for the local regional hub leaders to properly inform the public about the cohort. This proposed Program Office is a very successful model for Corona Immunitas as well. Professionals from project management, communication, law and logistics were engaged, and thereby enabled the scientists to focus on their core domain of research.

International Scientific Advisory Board—SAB (or Beirat): The sound implementation and operation of a large multi-centre cohort and biobank requires the coordination of a range of expertise. To strengthen the quality of such large-scale projects, scientists and the funders alike greatly profit from independent external scientific advice. Given the national character of the cohort project and the small size of Switzerland, it will be extremely difficult to identify leading experts in Switzerland that are not part of the cohort themselves. Thus, an international scientific advisory board is proposed instead. Internationally respected experts provide independent scientific advice such as on specific methods and work packages, including ethical-legal or social (ELS) aspects of the study in support of the Executive Board (EB) and the ELS-Work Package. A limited number of advisors will serve for 4 years. The EB may opt for *ad hoc* advisors to cover specific upcoming topics. In the early phase, SAB may be assembled once a year or as needed.

If coordinated by a strong Program Office, the Work Package structure is an efficient organizational model for the organization of the multi-dimensional research domains needed in large multi-centre cohort studies. Each work package (WP) covers well defined tasks and activities and the related methods and tools. Each WP has a scientific leader who is also a member of the Scientific Steering Committee.

Transversal Work Packages (WP) cover technical, scientific or logistic issues that are of relevance for the entire program or at least a range of vertical work packages. Advised by the Steering Committee, SSPH+ mandates WPs to institutional partners with the required expertise and commitment to collaborate and deliver and taking into account the governance of other Swiss research platforms with overlapping tasks and needs (e.g., the organizational bodies of the Swiss Personalized Health Network - SPHN). Prominent examples of cohort transversal work packages will be a WP Biobanking, WP ELS for Ethical, Legal and Social issues or WP Data Interoperability, WP Communication, or the WP Survey Tools and Translations for the development, testing and translation of core questionnaire tools. The EB may call for Transversal WP’s also to cover specific scientific avenues or themes such as, e.g., a WP Exposome, WP Mental Health, a WP Social Media and Health, a WP imaging, a WP omics analysis, a WP biostatistics. Scientifically focused WPs may also be coordinated by scientific experts from vertical WPs.

Vertical Work Packages cover in particular locally rooted research tasks and activities, such as in particular the running of the regional study centers. Those are leading academic institutes experienced with epidemiologic methodologies to conduct population based scientific field work. Thus, vertical WP will be instrumental in the collection of high-quality research data in local centers all across Switzerland. These hubs are led by leading public health researchers and institutions with a record in scientific field work in collaboration with participants.

Coordinated by the Program Office transversal and vertical WPs closely collaborate and interact. For example, whilst the WP Biobanking will provide all concepts and standard operating procedures for the collection of biomaterial, the vertical WPs of each region will reach out to the participants to take the collect, store and deliver the respective biospecimens. The number of WP’s may change over time, as needed.

### Funding

The cost for building and maintaining a long-term study and biobank with at least 100,000 participants will surpass CHF 100 million in the long term and depend on matching in-kind and cash contributions from Swiss research institutes. This estimate reflects experience from other cohorts abroad and the costs for the multi-centric SAPALDIA cohort and biobank in Switzerland, extrapolated to 100,000 participants. The costs do not cover all relevant–omics analysis or whole genome sequencing of all participants, where additional funding sources will be needed, including private funding.

The estimated investments are expected to provide the necessary critical mass for numerous highly complex processes, while ensuring optimal privacy protection, semantic interoperability and ethical standards. It allows a high number of samples and data points of comparable quality, handled according to the same protocol and stored in a secured way. It increases with the number of research projects and partnerships that can benefit from the resource. For example, the US All of Us research program is therefore, committed to engaging multiple sectors and forging strong partnerships with academic and other non-profit researchers, as well as patient groups and the private sector to capitalize on work already underway.

To assure a high return on investment a broad partner network must be involved in designing the cohort, bio sampling and imaging procedures. Partners for building and using data from a Swiss Cohort and Biobank range from basic research (e.g., genomics) to clinical research (all domains including rare disease/medical genetics; radiology, neurology), epidemiology and public health, health economics, toxicology and the social sciences. In addition to academic partners, various political bodies (e.g., FOPH, FOEN, FSVO, BASPO, SECO, BLV, BFU) rely on a Swiss-specific longitudinal evidence base for policy setting.

The first white paper for the implementation of the SPHN initiative has already documented in its basic report the mid-term need for a population cohort and reference. The SPHN data infrastructure currently being established to facilitate the use of patient data for research will greatly facilitate the identification of diagnosis incidence in a population cohort. Beyond that, setting up a population reference cohort in the next funding period would build on and leverage the investments made in SPHN. The White Paper for the SAMW clinical research platform points to the need for population cohort data. The Roadmap Infrastructure call clearly pointed to the need for Swiss Bio and Swiss Imaging Reference data.

Funding principles can be guided by the funding of very large cohorts abroad. The **NAKO** multicenter project is funded by the Federal Ministry of Education and Research (Bundesministerium für Bildung und Forschung, BMBF), the participating federal states and the Helmholtz Association. **UK Biobank** is funded primarily by the “Wellcome” charity and the Medical Research Council (MRC). Both organizations have provided funds to plan, roll out and maintain the study, and to enhance the resource as the study has matured. Some companies have invested considerably to analyze data and to make those new results available to the wider research community. For instance, a consortium of companies led by Regeneron in the US is undertaking exome sequencing of UK Biobank genetics data at a cost to them of many millions of US dollars. On other occasions, companies have provided research platforms to UK Biobank at significant discounts in order to accelerate the accumulation of data available to researchers on UK Biobank participants.^29^ The **CONSTANCES** cohort is supported by the Caisse Nationale d’Assurance Maladie des travailleurs salariés-CNAMTS, and was funded in its pilot phase by the “Direction générale de la santé” of the Ministry of Health, and by the Institut de Recherche en Santé Publique-Institut Thématique Santé Publique, and the following sponsors: Ministère de la santé et des sports, Ministère délégué à la recherche, Institut national de la santé et de la recherche médicale, Institut national du cancer et Caisse nationale de solidarité pour l’autonomie (AMC10003LSA) 104. **LifeGene** is funded by public-private partnership.

**Funding of the large-scale Swiss Cohort and Biobank is therefore timely and should take the following aspects into consideration:**

• Sustainable public funding of SSPH+, the research hubs and of data collection for a period of at least 10 years of follow-up, with the possibility for renewal of funding thereafter.
• Funding as a research infrastructure and coordinated by researchers to allow continuous and efficient adaptation of the research protocol to upcoming research priorities without legal restrictions.
• Allowing for the possibility of integrating unrestricted private funds following the principle of the Corona Immunitas research program. A work package for public-private partnership integrated into the Roadmap Infrastructure project IOP4CH is foreseen to develop modes for public-private partnership collaboration in the context of cohort.
• Public-private funding of–omics analyses in large number of samples to avoid bias arising from batch effects and sample wasting.

## Editorial Note

This Society Statement is published for the Swiss School of Public Health (SSPH+), a foundation carried by twelve Swiss universities, and the Swiss Society of Public Health. SSPH+ is responsible and liable for all contents of this Society Statement. It was not peer-reviewed by PHR editorial processes but in a public process (see Acknowledgement).

## Footnotes

1
https://www.parlament.ch/de/ratsbetrieb/suche-curia-vista/geschaeft?AffairId=20194069

2
https://www.parlament.ch/de/ratsbetrieb/suche-curia-vista/geschaeft?AffairId=20213220

3
https://www.healthaffairs.org/do/10.1377/forefront.20161121.057630

4
https://www.bfs.admin.ch/bfs/en/home/statistics/population/births-deaths/life-expectancy.html

5
https://m2rfilms.com/making-pandemics

6
https://www.ipcc.ch/2022/02/28/pr-wgii-ar6/

7
https://www.shcs.ch/

8
https://www.childhoodcancerregistry.ch

9
https://www.bag.admin.ch/bag/de/home/strategie-und-politik/gesundheit-2030/gesundheitspolitische-strategie-2030.html

10
https://trapego.ch/

11
https://www.hbm4eu.eu/

12
https://www.bag.admin.ch/bag/de/home/das-bag/aktuell/medienmitteilungen.msg-id-88132.html

13
https://nda.nih.gov/abcd/

14
https://www.swissnationalcohort.ch/

15
https://www.corona-immunitas.ch

16
https://www.cartagene.qc.ca/

17
https://childstudy.ca

18
https://www.growingup.ie

19
https://abcdstudy.org/

20
www.ihccglobal.org

21
https://smw.ch/index.php/smw/announcement/view/17

22
https://allofus.nih.gov

23
https://nako.de/

24
https://lifegene.se/

25
https://childstudy.ca/

26
http://www.bristol.ac.uk/alspac/

27
https://www.kiggs-studie.de/

28
https://abcdstudy.org/about/

29
https://www.ukbiobank.ac.uk/media/f32lged4/funding-uk-biobank-summary.pdf
